# Identification of a novel MSI-related ceRNA network for predicting the prognosis and immunotherapy response of gastric cancer

**DOI:** 10.18632/aging.204794

**Published:** 2023-06-12

**Authors:** Lei Zhang, Lu Cao, Jinqiang Liu, Lili Duan, Wei Zhou, Ting Li, Lei Guan, Xiaoming Wu, Huqin Zhang

**Affiliations:** 1 Key Laboratory of Biomedical Information Engineering of Ministry of Education, School of Life Science and Technology, Xi’an Jiaotong University, Xi’an 710032, Shaanxi Province, China; 2 Department of Biomedical Engineering, Air Force Hospital of Eastern Theater Command, Nanjing 210001, Jiangsu Province, China; 3 State Key Laboratory of Cancer Biology and National Clinical Research Center for Digestive Diseases, Xijing Hospital of Digestive Diseases, Fourth Military Medical University, Xi’an 710032, Shaanxi Province, China

**Keywords:** gastric cancer, microsatellite instability

## Abstract

Background: Mounting evidence has underscored the pivotal role of the competitive endogenous RNA (ceRNA) regulatory networks among various cancers. However, the behavior characteristics and complexity of the ceRNA network in Gastric cancer (GC) remains unclear. In this study, we aimed to clarify a Microsatellite instability (MSI)-related ceRNA regulatory network and identify potential prognostic markers associated with GC.

Methods and Results: We extracted transcriptome data of GC patients from The Cancer Genome Atlas (TCGA) and identified differentially expressed lncRNAs, miRNAs and mRNAs based on MSI status. A hub ceRNA network including 1 lncRNAs (MIR99AHG), 2 miRNAs and 26 mRNAs specific to MSI was established in GC. We further constructed a prognostic model with seven target mRNAs by Lasso Cox regression, which yielded AUC values of 0.76. The prognostic model was further validated in an external independent dataset that integrated three GEO datasets. The characterization of immune cell infiltration and immunotherapy effects between high-risk and low-risk groups were then analyzed. Immune cell infiltration was significantly different between high- and low-risk groups based on risk scores. GC patients with lower risk scores correlated with better immune checkpoint inhibitor therapy (ICI) response. We further validated the expression and regulatory relationship of the ceRNA network *in vitro* experiments, and also confirmed the relationship between MIR99AHG and PD-L1.

Conclusions: Our research provides in-depth insights on the role of MSI-related ceRNA in GC and the prognosis and ICIs therapy response of GC patients can be assessed by the risk model based on MSI-related ceRNA network.

## INTRODUCTION

Gastric cancer (GC) is one of the most common and fourth most lethal malignant cancers worldwide [1]. Unfortunately, patients with GC are often diagnosed at advanced stages and have few effective treatment options, resulting in poor prognosis [2]. Therefore, novel therapeutic approaches and biomarkers are urgently needed to improve the prognosis of gastric cancer. In 2014, The Cancer Genome Atlas (TCGA) proposed 4 molecular GC subtypes, including microsatellite instability (MSI), which accounts for about 22% of GC cases [3]. According to existing literature, MSI is caused by failure in the DNA mismatch repair system, and is characterized by accelerated accumulation of mutated single nucleotides, which further results in alterations in the length of microsatellite sequences that are prevalent in the genome [4]. Several MSI studies on GC have shown that the overall survival of patients in the MSI group was better than that in the stable category, for participants who had received surgical resection alone or a combination of surgery and radiotherapy. However, this trend is inconsistent in patients receiving chemotherapy [3]. Additionally, recent studies indicated that GC patients with MSI are more likely to benefit from immune checkpoint inhibitors (ICIs) [5]. Therefore, further attention should be paid to how MSI affects the prognosis of GC and the efficacy of immunotherapy.

The Competing endogenous RNA network (ceRNA network) is a ubiquitous regulatory network, which greatly enriches the genetic diversity of the human genome [6]. In traditional conditions, miRNAs exert their function of regulating gene expression by binding to the mRNAs’ miRNA response elements (MREs), and further guiding the Argonaute protein to activate the degradation of target genes. However, recent insights propose that miRNAs can be competitively absorbed by other long non-coding RNAs (lncRNAs) containing the same MREs, thus affecting their regulatory effect on target genes. This type of lncRNAs is called ceRNAs and the wide crosstalk among genes mediated by miRNAs is referred to the ceRNA network [7]. Because the same MREs can be contained in many different lncRNAs and target genes, the formed ceRNA regulatory network is very large and nearly 60% of coding genes are regulated by this kind of crosstalk [7]. Recent studies have shown that ceRNA networks also play a critical role in the progression of GC [8]. Some lncRNAs, for example *HULC, RP11-314B1.2, LINC00106 and RP11-999E24.3* can affect the prognosis of GC patients through the ceRNA mechanism, presenting potentially new therapeutic targets [8]. Furthermore, several studies have shown that the ceRNA network may be related to immune cell infiltration in the tumor microenvironment (TME) [9]. However, to the best of our knowledge, there are no studies on the ceRNA network in the MSI GC subtype, and it is therefore necessary to explore the possible ceRNA regulatory network under microsatellite instability and its impact on the prognosis of GC.

Over the recent years, immune checkpoint inhibitor (ICI) therapy has gained popularity as a new form of cancer treatment. In 2017, the United States (US) Food and Drug Administration (FDA) approved the use of pembrolizumab for the treatment of PD-L1+ GC patients. Additionally, a retrospective analysis also confirmed that patients with MSI are more likely to benefit from ICI [3]. However, some patients still have little response to ICIs therapy or exhibit resistance to ICIs [10]. Therefore, it is urgent to find novel biomarkers that can predict and improve ICI efficacy and prognosis of GC.

In this study, differentially expressed mRNAs, miRNAs and lncRNAs (DEmRNAs, DEmiRNAs, DElncRNAs) between patients with high microsatellite instability (MSIH) and microsatellite stability or low microsatellite instability (MSIL/S) were analyzed. The MSI-related ceRNA network was established using the “GDCRNATools” package in R. In addition, survival analysis and location prediction were conducted targeting the lncRNAs in the network and MIR99AHG, located in the cytoplasm, was identified as a hub lncRNA with prognostic significance. MIR99AHG encodes lncRNA MIR99AHG as well as a *miR-99a/let-7c/miR-125b2* cluster on chromosome 21q. Precious studies reported that MIR99AHG was involved in progression of head and neck squamous cell carcinoma and lung squamous cell carcinoma, and correlated with survival rate [11, 12]. In addition, MIR99AHG could inhibited glioblastoma temozolomide sensitivity [13]. Of note, MIR99AHG could accelerate EMT and suppress apoptosis of GC cells by miR577/FOXP1 axis [14]. The specific mechanism of MIR99AHG in the progression of GC and its relationship with immunotherapy still need further exploration. Therefore, the MIR99AHG-related subnetwork was screened and nodes with prognostic value were retained. The prognosis prediction model based on this network was then established using TCGA data and validated in independent GEO datasets. Next, correlation between our predicted risk score and ICI efficacy was analyzed. The results showed that our predictor could reflect patients’ responses to ICI to a certain extent. Finally, differences in immune cell infiltration and expression of chemokine genes between different risk groups were simultaneously analyzed. The co-expression patterns of these immune cells and chemokines were also identified and their relationship with MIR99AHG analyzed. The MSI-related ceRNA network established in this study provides new insights on the mechanisms through which MSI regulates GC progression and influences prognosis. Moreover, the generated GC-prognostic model based on the hub network can successfully predict the prognosis of patients and forecast response to ICI.

## RESULTS

### Differentially expressed mRNAs (DEmRNAs), miRNA (DEmiRNAs) and lncRNAs (DElncRNAs) between MSIH and MSIL/S

**Figure 1:**
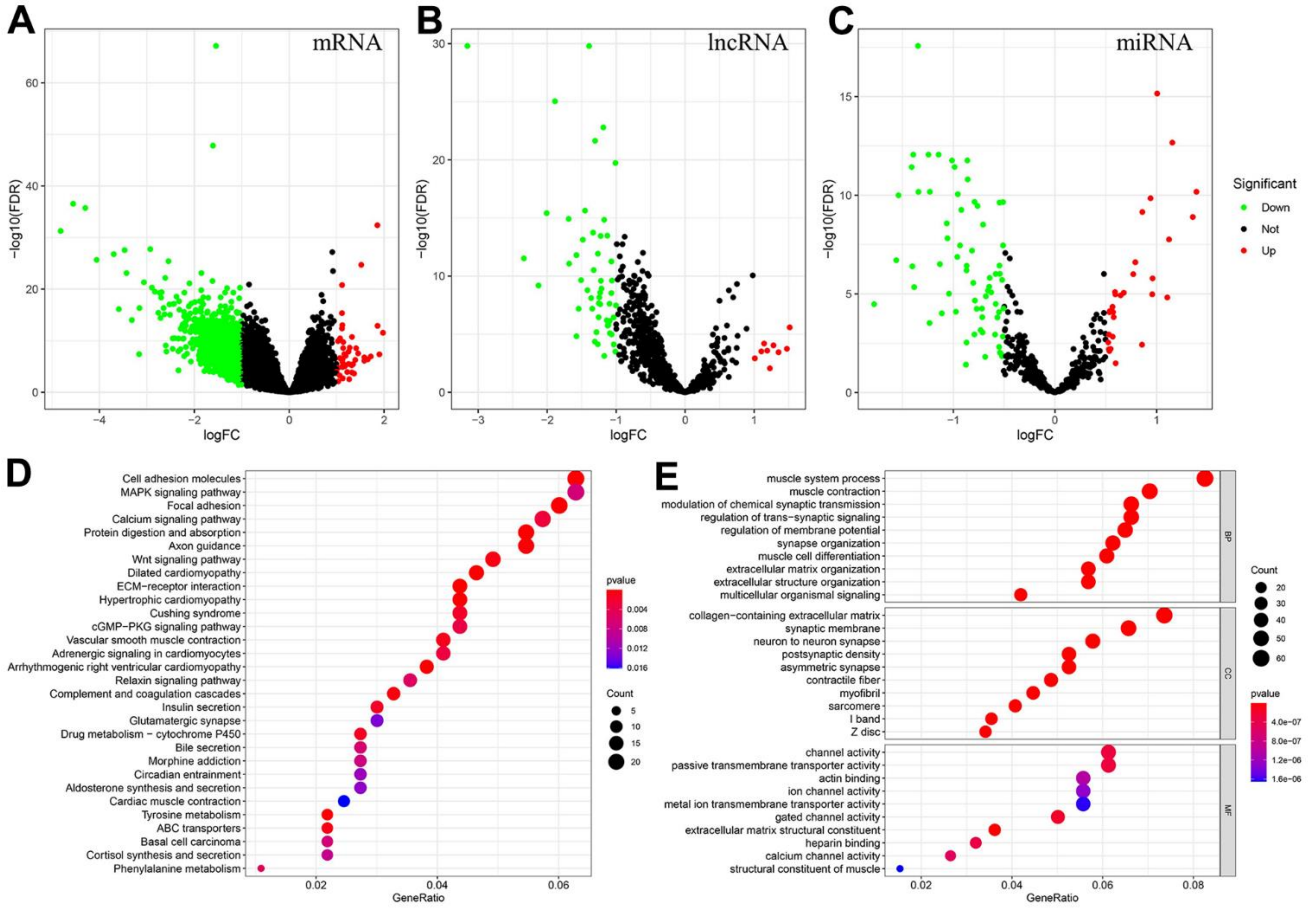
**Identification of DEmRNAs, DEmiRNAs, DElncRNAs, and enrichment analysis of genes associated with the MSI-related ceRNA network.** (**A**–**C**) DEmRNAs, DElncRNAs and DEmiRNAs between MSIH and MSIL/S. (Red represents upregulated whereas green represents downregulated genes). (**D**, **E**) The KEGG and GO enrichment analysis of genes in the MSI-related ceRNA. (Based on the gene ratio, the top 30 KEGG pathways and top 10 GO terms are shown).

Supplementary Table 1 shows the patient information collected in this study, and the proportion of patients with different clinical characteristics. DEmRNAs, DEmiRNAs and DElncRNAs between the MSIH and MSIL/S groups were analyzed based on the 343 samples with MSI information from TCGA-STAD. The screening conditions were set at |log2 (fold change) |>1 and FDR<0.05 for mRNA and lncRNA, and |log2 (fold change) |>0.5 and FDR<0.05 for miRNA. The results in Figure 1A–1C show that there were 900 DEmRNAs of which 44 and 856 were up and down regulated, respectively; 96 DEmiRNAs of which 29 and 67 were up and down regulated, respectively; and 60 DElncRNAs of which with 9 and 51 were up and down regulated, respectively.

### Construction of an MSI-related lncRNA-miRNA-mRNA ceRNA network

Based on the screened DEmRNAs, DEmiRNAs and DElncRNAs, the “gdcCEAnalysis” function in the “GDCRNATools” package was used to construct the MSI-related ceRNA network. The cut-off P values for the hypergeometric and Pearson correlation tests were both set at 0.01 and the regulation similarity value was required to be higher than 0. The constructed ceRNA network consisting of 2,729 lncRNA-mRNA pairs, 13,334 edges and 895 nodes (808mRNA, 44 miRNAs and 43 lncRNAs), is shown in Supplementary Table 2. The table also contains information on LncRNA-miRNA-mRNA pairs, hypergeometric distribution, correlation test details, regulation similarity and sensitivity correlation. Enrichment analysis was conducted based on the 808 mRNAs. The findings in Figure 1D, 1E show that 44 KEGG pathways were enriched, including MAPK signaling pathway, focal adhesion and Wnt signaling pathway. On the other hand, GO analysis revealed that the most enriched biological process (BP) was the muscle system process, the most enriched cellular component (CC) was the collagen-containing extracellular matrix, and the most enriched molecular function (MF) was channel activity.

### Screening of the hub MSI-related ceRNA with prognostic significance

**Figure 2:**
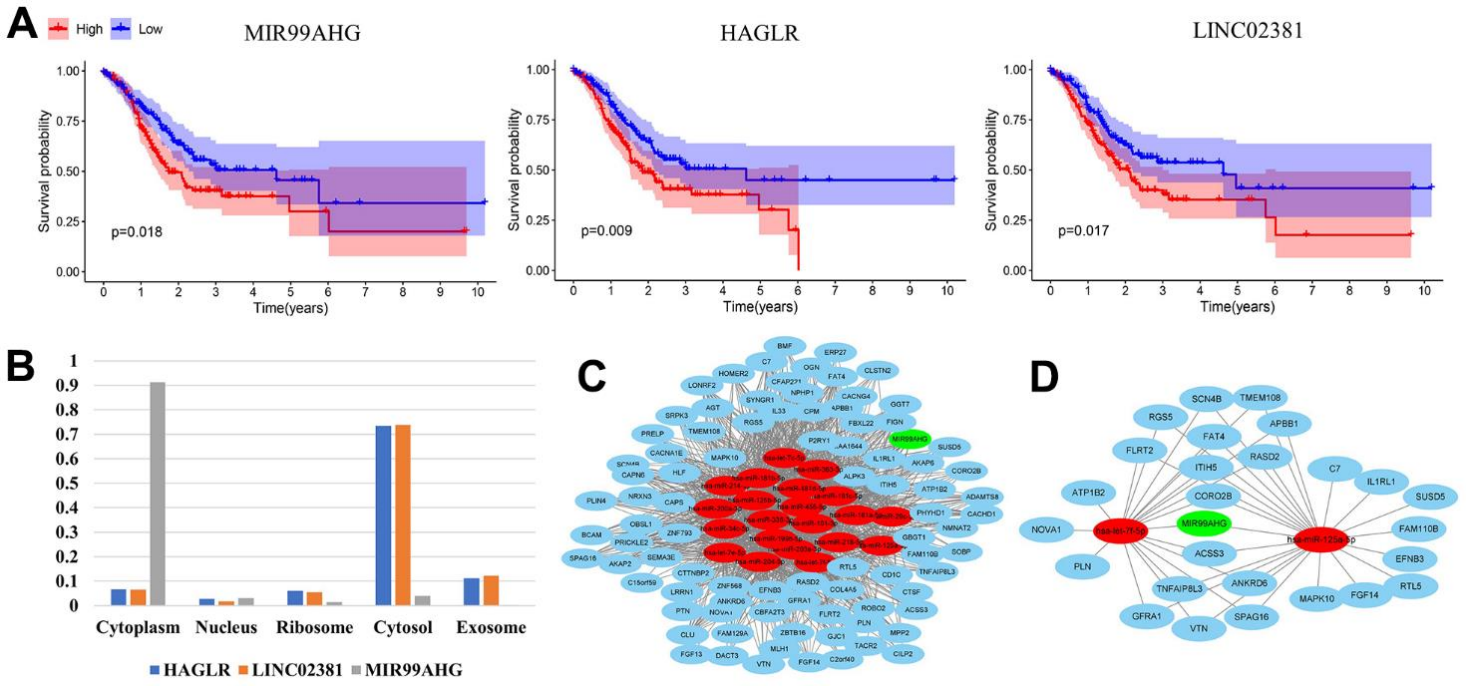
**Construction of the final hub ceRNA network.** (**A**) Three lncRNAs with prognostic significance: MIR99AHG, HAGLR and LINC02381. (**B**) Predicted location of MIR99AHG, HAGLR and LINC02381. (**C**) MIR99AHG-related ceRNA network. (**D**) The final hub ceRNA network with prognostic significance. (In the above network, green, red and blue circles represent lncRNA, miRNA and mRNA, respectively).

We conducted survival analysis on 43 lncRNAs in the constructed MSI-related ceRNA network. Three lncRNAs with prognostic significance were identified, including *MIR99AHG, HAGLR and LINC02381* (Figure 2A). Based on the sequences downloaded from NCBI, the lncLocator prediction results (Figure 2B) showed that only MIR99AHG was located in cytoplasm. Therefore, MIR99AHG was selected as the hub lncRNA and the MIR99AHG-related ceRNA network was shown in Figure 2C. This subnetwork contained 87 lncRNA-mRNA pairs, 1116 edges and 109 nodes (1 lncRNA, 21 miRNAs and 87 mRNAs). Additionally, Unicox analysis was conducted, targeting the nodes in this subnetwork (Supplementary Table 3). Only nodes with prognostic significance were retained for constructing the final hub ceRNA network (Figure 2D). This network is comprised of 26 lncRNA-mRNA pairs, 43 edges and 29 nodes (1 lncRNA, 2 miRNA and 26 mRNA). Of the two miRNAs, let-7f-5p was identified as a protective factor while mir-125a-5p was shown to be the risk factor. All the 26 mRNAs were risk-associated genes.

### Establishment and validation of the prognosis prediction model based on the MSI-related ceRNA regulatory network

**Figure 3:**
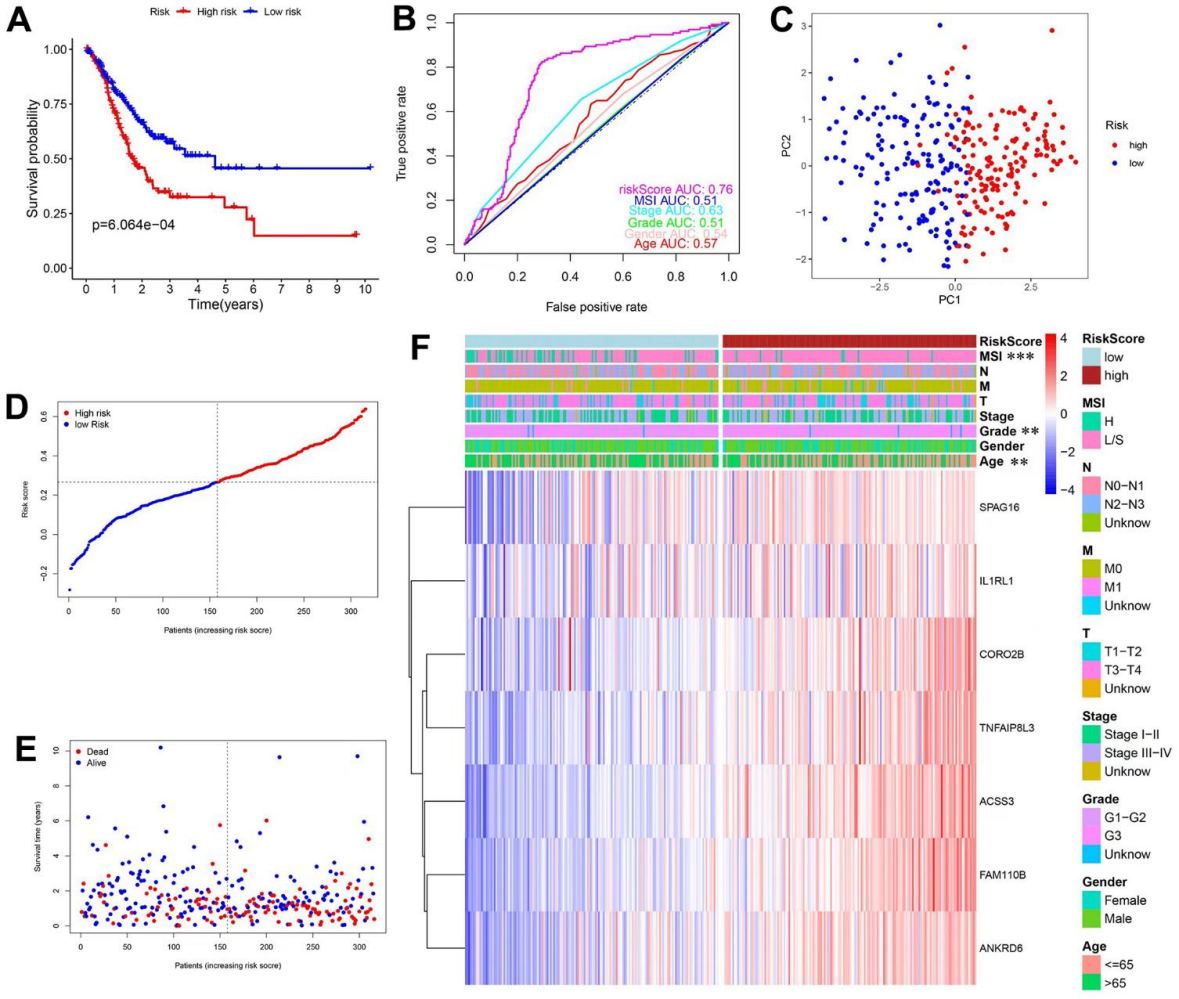
**Construction and validation of prediction model in the TCGA database.** (**A**) KM curves and survival analysis. (**B**) Time-dependent ROC curves and AUCs. (**C**) PCA analysis. (**D**) Distribution of risk scores and median values. (**E**) Scatter plot showing the overall survival of patients. (**F**) Heatmap of the 7 genes used in the model.

The Lasso Cox regression algorithm was applied to establish the prognosis prediction model, based on the 26 mRNAs in the final hub ceRNA network (Supplementary Figure 2). The constructed model comprised of 7 genes and the risk score was calculated as follows: RiskScore=IL1RL1*0.042604+SPAG16*0.069518+FAM110B*0.050744+ANKRD6*0.017904+ACSS3*0.068896+CORO2B*0.034964+TNFAIP8L3*0.036276. Patients were stratified into the high- and low-risk groups based on the predicted score. In addition, KM curves were plotted, and survival difference was analyzed. The results revealed that high-risk patients had worse OS (Figure 3A). Moreover, ROC curve analysis (Figure 3B) showed that the AUC of the prediction model was 0.76. Results from PCA (Figure 3E) showed that the distribution of these 2 groups was diverse, and could not be separated linearly. Moreover, the scatter plots (Figure 3E) showed that, with increased risk scores, patients had a shorter lifespan and a higher possibility of death. Finally, a heatmap was used to show the expression patterns of the 7 genes used in the model. As shown in Figure 3F, all the genes were risk factors for GC patients. Additionally, the precited risk score was associated with some clinical characteristics, therefore correlation tests were conducted. The findings revealed that a high risk was associated with MSIL/S, a higher grade and a younger age (Supplementary Figure 3A).

**Figure 4:**
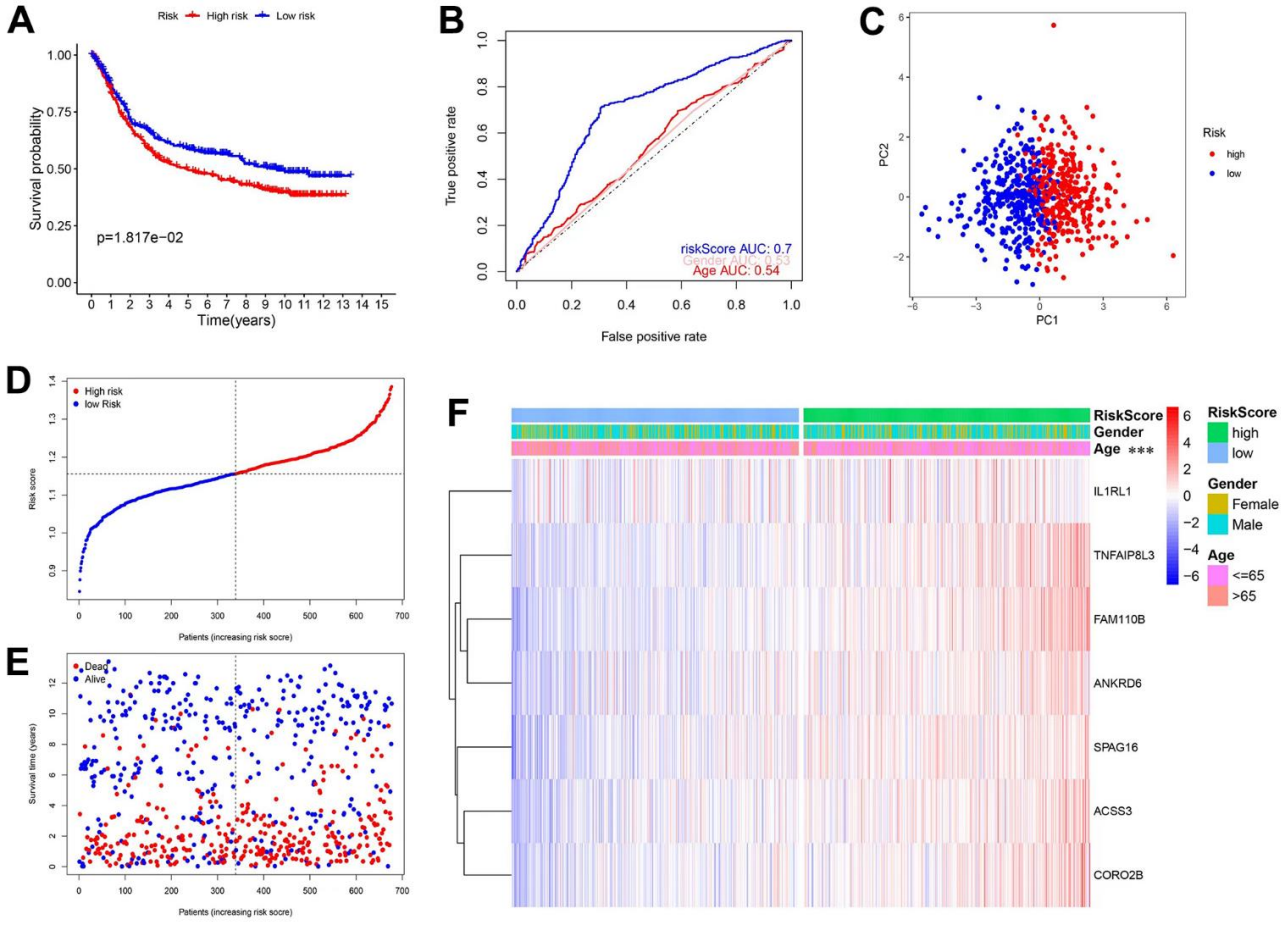
**Validation of the prediction model in the GEO database.** (**A**) KM curves and survival analysis. (**B**) Time-dependent ROC curves and AUCs. (**C**) PCA analysis. (**D**) The distribution of risk scores and median values. (**E**) Scatter plot showing the overall survival of patients. (**F**) Heatmap of the 7 genes used in the model.

To test the generalization ability of the established model, the above tests were repeated in an independent GEO dataset (n=689). The overall results were consistent with those obtained from the TCGA database, further validating our model. Figure 4A shows that high-risk patients had worse OS and Figure 4B shows that the AUC was 0.7. The PCA results (Figure 4C) showed that the distribution of the 2 groups was different and the scatter plot (Figure 4E) confirmed that the mortality rate increased with a higher risk score. Finally, the heatmap showed that the 7 genes were all risk factors, and a higher risk was associated with a younger age (Supplementary Figure 3B).

**Figure 5:**
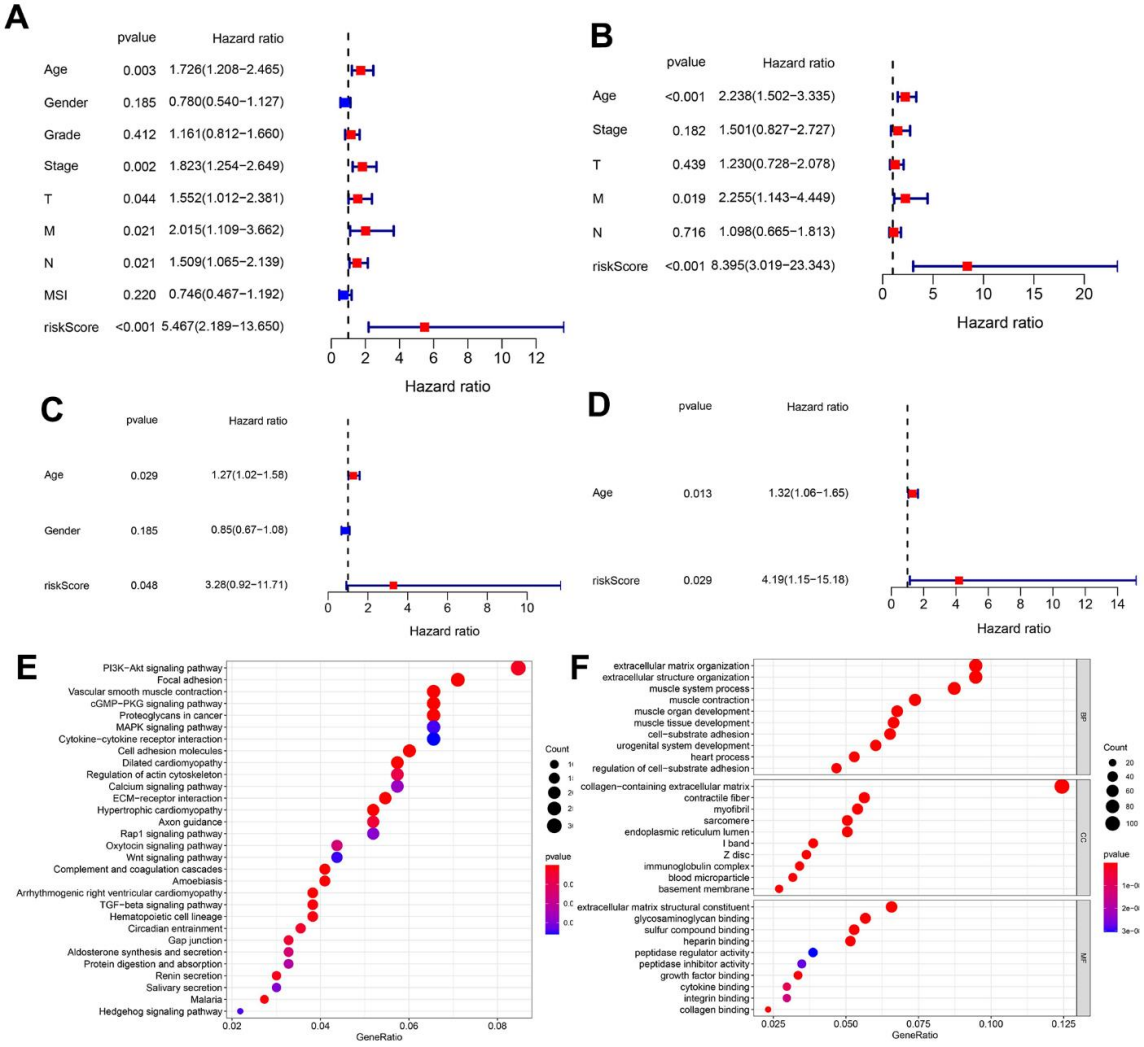
**Unicox and multicox analyses, and enrichment analysis targeting the DEmRNAs in different risk groups.** (**A**, **B**) Unicox and Multicox analyses in the TCGA database. (**C**, **D**) Unicox and Multicox analyses in the GEO database. (**E**, **F**) KEGG and GO analyses targeting DEmRNAs in different risk groups (According to gene ratio, the top 30 KEGG pathways and the top 10 GO terms are displayed for each category).

To further test whether the predicted risk score was an independent prognosis index for GC patients, this study integrated the risk score with other clinical factors into Unicox and Multicox analyses. Results for the TCGA database are shown in Figure 5A, 5B. Unicox analysis revealed that age, stage, T, M, N, and the risk score, were risk factors for GC patients (Figure 5A). On the other hand, Multicox analysis confirmed that age, M and risk score, were independent prognostic factors for GC (Figure 5B). Results for the GEO database were shown in Figure 5C, 5D. Unicox analysis showed that age and risk score were risk factors for GC patients (Figure 5C). Moreover, Multicox analysis identified these 2 factors (age and risk score) as independent prognostic factors for GC (Figure 5D). Furthermore, Differential expression gene (DEG) analysis was conducted in the TCGA database between the high- and low-risk groups. The results showed that 913 genes were up regulated in the high-risk group while 30 genes were downregulated. KEGG and GO enrichment analyses were then conducted on these DEmRNAs. A total of 52 pathways (Figure 5E) were enriched in the KEGG analysis and the most interesting included the PI3K-Akt signaling pathway, focal adhesion and vascular smooth muscle contraction. After comparing these pathways with the 43 enriched pathways in Figure 1D (representing the MSI-related ceRNA regulatory network), 28 pathways were shown to be conserved (Supplementary Table 4). This demonstrated that the constructed model generally represented the MSI-related ceRNA regulatory network. Finally, GO analysis showed that the most enriched BP, CC, and MF were extracellular matrix organization, collagen-containing extracellular matrix and the extracellular matrix structural constituent, respectively (Figure 5F).

### Correlation between risk score and IPS

**Figure 6:**
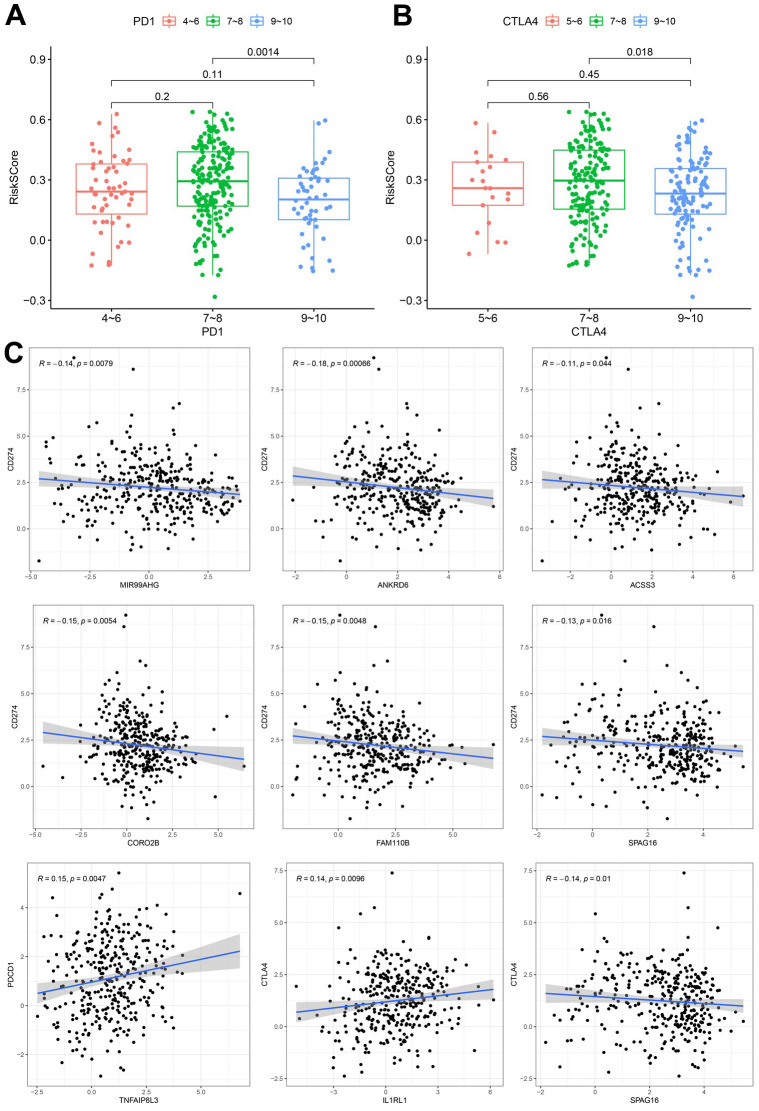
**Correlation between the constructed model and ICI.** (**A**) Analysis of risk scores among different IPS-PD1/PDL1/PDL2 groups. (**B**) Comparation of risk scores in the indicated IPS-CTLA4 groups. (**C**) Expression correlation between hub nodes and immune checkpoint.

315 IPS scores for TCGA-STAD patients were downloaded from the TCIA database and integrated into patient data. The IPS score can be classified into 2 types, where one type represents the possibility of response to the PD1/PDL1/PDL2 blocker, while the other represents the possibility of response to the CTLA4 blocker. Additionally, patients can be divided into 3 groups based on the IPS score: 4-6, 7-8 and 9-10. With respect to the PD1/PDL1/PDL2 blocker, these 3 groups of patients accounted for 18%, 66% and 16%, respectively, while for the CTLA4 blocker, these 3 categories of patients accounted for 7%, 57% and 36%, respectively. Differences in risk scores among the 3 groups were compared and the results are shown in Figure 6A, 6B. In both therapies, the risk score of the 9-10 group was significantly lower than that of the 7-8 category, indicating that a lower risk score was associated with a better ICI response. These results demonstrated that our calculated signature could not only predict OS but also forecast the ICI response to a certain degree. To further explore this interesting observation, we assessed the correlation between the expression of the hub nodes (MIR99AHG and 7 genes used in the model) and 3 immune checkpoint genes (*PD-L1(CD274), PD1(PDCD1), CTLA4*). The findings showed that *MIR99AHG*, *ANKRD6*, *ACSS3*, *CORO2B*, *FAM110B*, and *SPAG16* were negatively associated with PD-L1 (Figure 6C). Additionally, *TNFAIP8L3* was positively correlated with PD1, while *IL1RL1* was positively correlated with CTLA4. However, *SPAG16* was negatively correlated with CTLA4.

### Analysis of immune cell infiltration between different risk groups

This study simultaneously investigated immune cell infiltration and chemokine gene expression among different risk groups in the TCGA and GEO databases. The results revealed that there were 8 immune cells with consistent infiltration in the 2 databases (Figure 7A, 7B). T cells CD4 memory resting, Mast cells resting, B cells naïve and Monocytes were infiltrated in the high-risk group, while T cells CD4 memory activated, Mast cells activated, Neutrophils and Macrophages M1 were mainly infiltrated in the low-risk group. In addition, the stromal score and the estimate score in the high-risk group were markedly higher, demonstrating that the patients had lower tumor purity. And the risk score was positively correlated with the stromal score and the immune score (Supplementary Figure 4). Additionally, 17 chemokines were consistently expressed in the 2 databases (Figure 7C, 7D). A total of 14 chemokines (XCL1, CCL2, CCL11, CCL13, CCL16, CCL17, CCL19, CCL21, CCL22, CCL23, CXCL12, CXCL13, CXCL14 and CX3CL1) were highly expressed in the high-risk group, while three others (CCL20, CXCL1 and CXCL2) were mainly expressed in the low-risk group. The correlation of the above immune cells of interest with chemokines was further studied. Eight co-expression patterns were identified among 5 immune cells and 6 chemokines (Co-expression patterns were denoted as “chemokine ~ immune cell”), as shown in Figure 7E, 7F. They included CX3CL1 ~ T cells CD4 memory activated, CXCL14 ~ B cells naïve, CXCL12 ~ mast cells resting, CXCL1 ~ neutrophils, CXCL2 ~ neutrophils, CXCL1 ~ mast cells activated, CXCL2 ~ mast cells activated and CCL19 ~ B cells naïve. This study also investigated the association between the hub lncRNA MIR99AHG and the 6 identified chemokines. MIR99AHG was observed to be positively related to CXCL14, CXCL12 and CCL19, but negatively correlated to CX3CL1, CXCL1 and CXCL2 (Figure 7G). Finally, survival analyses were performed on the five identified immune cells in the 2 databases. The results showed that Mast cells resting and T cells CD4 memory activated were significantly correlated with the OS of patients in the GEO database (Figure 7H). Moreover, Mast cells resting was identified as a risk factor while T cells CD4 memory activated had the opposite effect.

**Figure 7:**
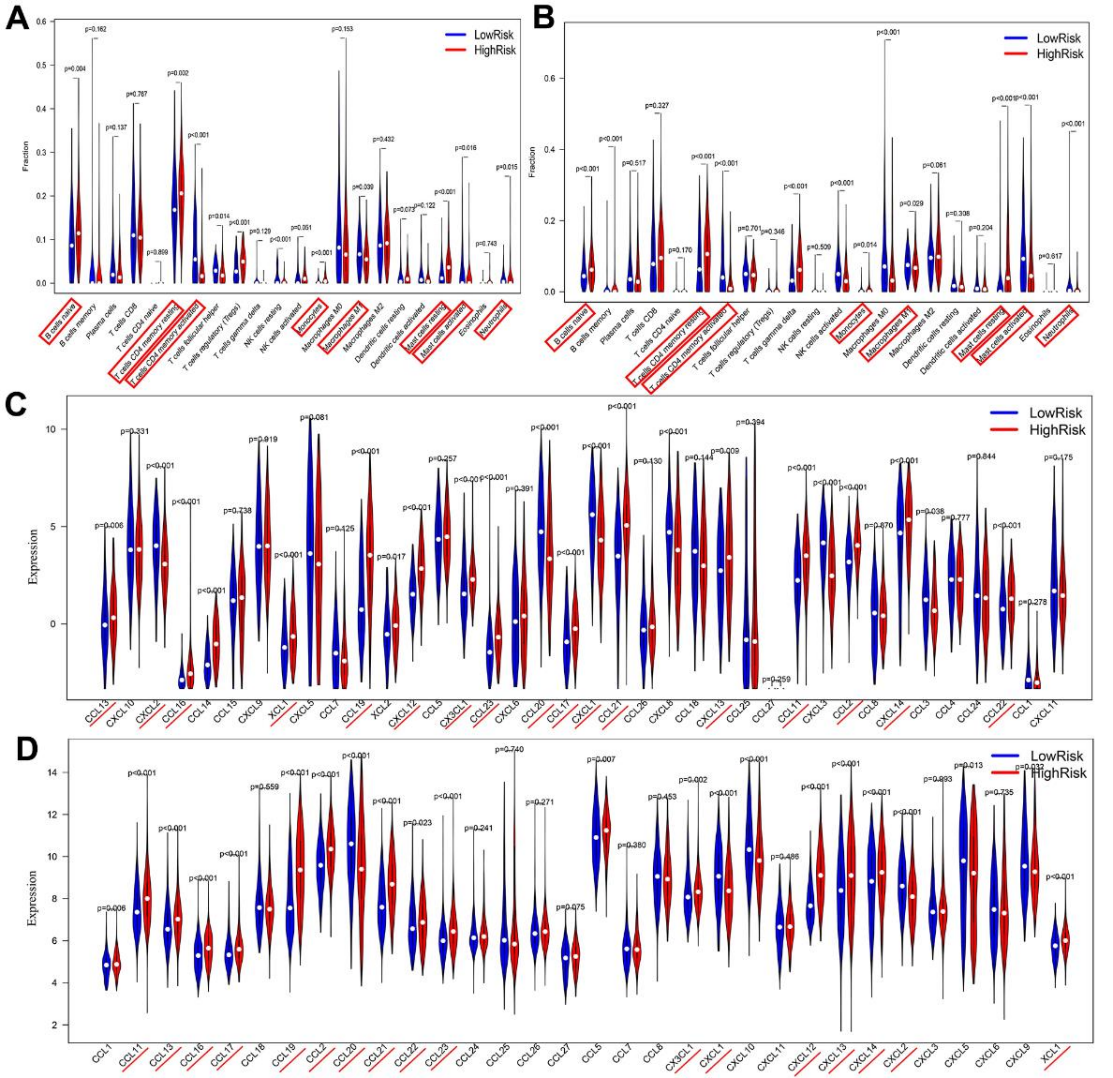
**Correlations among risk score, immune cell infiltration and chemokine expression.** (**A**, **B**) Differential infiltration of immune cells between high- and low-risk groups in the TCGA and GEO databases. (**C**, **D**) Differentially expressed chemokines between high- and low-risk groups in TCGA and GEO databases..

**Figure 7:**
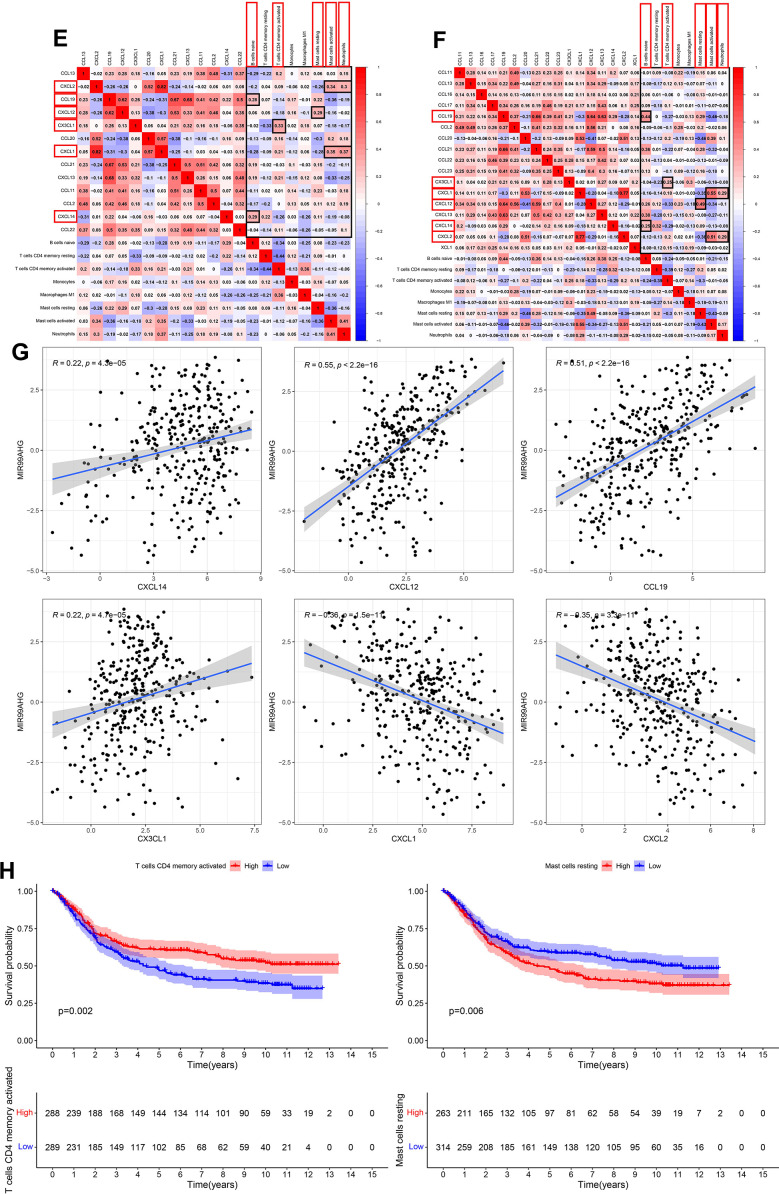
**Correlations among risk score, immune cell infiltration and chemokine expression.** (**E**, **F**) Co-expression pattern of immune cells and chemokines in TCGA and GEO databases. (Red box represents consistent trend in both databases) (**G**) Correlation between MIR99AHG and chemokines of interest. (**H**) Survival analysis of the selected immune cells.

### Validation of the expression and regulatory relationship of the ceRNA network

**Figure 8:**
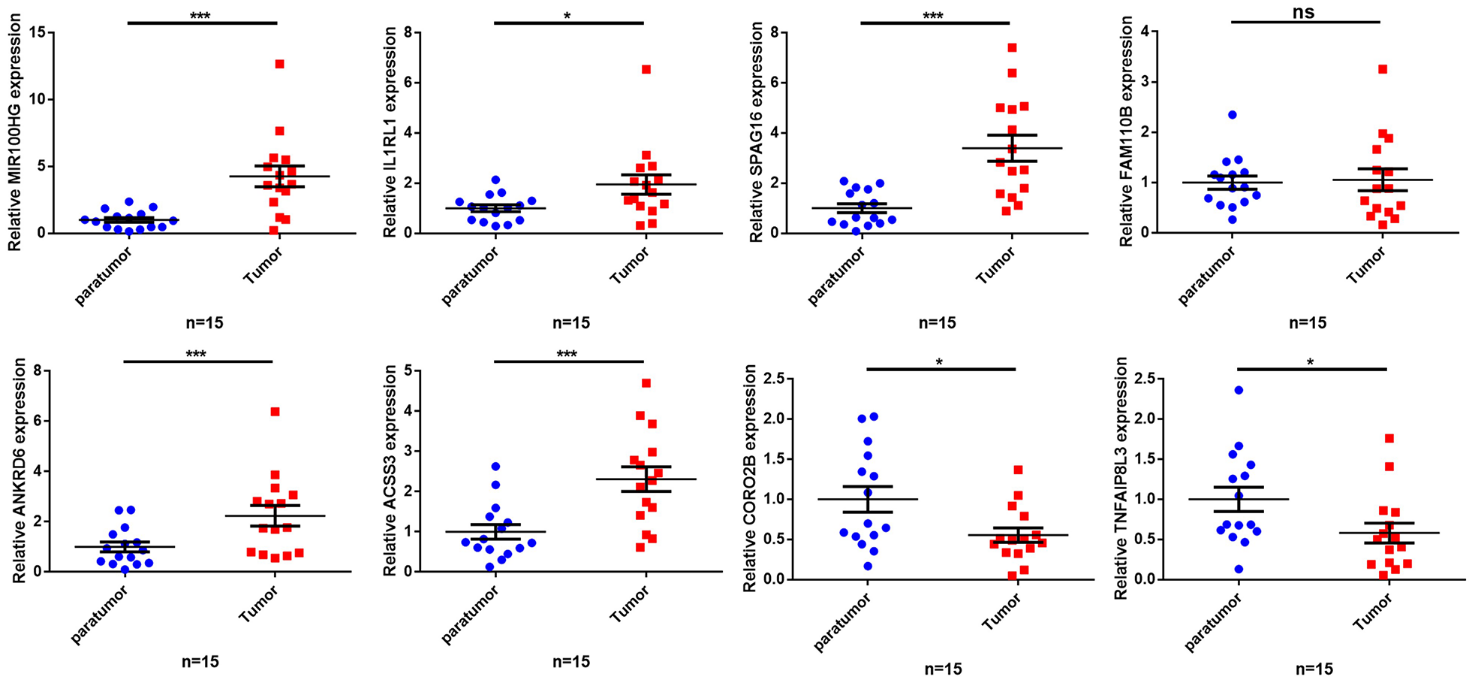
**Validation of the expression of key genes in human tissues.** The qRT-PCR results showing expression level of the 8 key genes. *p< 0.05, **p < 0.01, ***p < 0.001, ns: no significant difference.

To further validate our findings, we quantified the expression levels of MIR99AHG and the crucial genes in the prognostic model (*IL1RL1, SPAG16, FAM110B, ANKRD6, ACSS3, CORO2B and TNFAIP8L3*) in 15 GC samples and their adjacent nontumor tissues. The PCR results revealed that MIR99AHG, IL1RL1, SPAG16, ANKRD6 and ACSS3 were upregulated in GC tissues, while CORO2B and TNFAIP8L3 were downregulated (Figure 8). There was no significant difference in the expression of FAM110B between GC samples and the corresponding nontumor tissues. Due to the limited number of high-MSI samples, additional MSIH samples are needed to verify the expression levels of the above genes in different MSI types.

What’s more, we confirmed that MIR99AHG was overexpressed in GC cell lines compared with normal gastric epithelial cell (Supplementary Figure 5A). Knockdown or overexpression of MIR99AHG could negatively regulate the expression level of let-7f-5p and miR-125a-5p, and positively regulate the expression level of *IL1RL1, SPAG16, FAM110B, ANKRD6, ACSS3, CORO2B and TNFAIP8L3* (Supplementary Figure 5B, 5C)*.* The above results confirmed the regulatory relationship among the ceRNA network molecules. In addition, we analyzed the effect of MIR99AHG knockdown and overexpression on PD-L1 mRNA and protein expression, and also confirmed that MIR99AHG could negatively modulate PD-L1 expression (Supplementary Figure 5D–5F). The specific mechanism needs to be further elaborated.

## DISCUSSION

Globally, gastric cancer ranks fourth among the most common malignancies with poor prognosis [1]. As a crucial subtype of GC, MSI was shown to be associated with prognosis and ICI response [5]. On the other hand, ceRNA is a ubiquitous regulatory network in the human genome and is correlated with carcinogenesis, tumor progression and immune cell infiltration in the TME [7]. In this study, an MSI-related ceRNA network was, for the first time, constructed in GC, and a hub MIR99AHG-related subnetwork with prognostic significance was screened. The results showed that the prognosis model based on this network could successfully predict OS and was also related to ICI efficacy as well as immune cell infiltration in the TME.

MIR99AHG (also referred to as MONK) is located in chromosome 21q21.1, with the main function of promoting cell proliferation and differentiation. A previous study reported that MIR99AHG was significantly overexpressed in human GC tissues and can promote tumor progression. It was also identified as a significant risk factor for patient prognosis. Mechanistically, MIR99AHG acted as a ceRNA for mir-577, further activating the FOXP1-regulated Wnt/β-catenin pathway [15]. Additionally, Yutong Ma et al. classified MIR99AHG as an immune-associated lncRNA, which is significantly associated with survival in human skin melanoma (SKCM) and was used in the construction of a SKCM prognostic model [16]. Our study identified MIR99AHG as a risk factor for GC prognosis, consistent with previous findings. Interestingly, we also observed low expression of MIR99AHG in GC with MSI and its expression had a significant negative correlation with PD-L1 expression. Notably, most gene targets (*ANKRD6 ACSS3, CORO2B, FAM110B* and *SPAG16*) for MIR99AHG were also negatively correlated with PD-L1 and CTLA4 expression. According to results from Cox analysis, all the target genes and MIR99AHG were risk factors and had lower expression levels in the low-risk group. This trend was consistent with the observation that a lower risk score was correlated with higher expression levels of PD-L1 and a higher possibility of ICI response. These results suggest that the final ceRNA network may be involved in regulating the expression of immune checkpoint targets, thus affecting the efficacy of ICI in GC patients. Previous research also showed that immune checkpoint-inhibitor therapy alone may be inadequate, with a response rate of about 12%. Therefore, incorporating other molecular targets to improve the efficacy of ICI is an important aspect t that needs to be considered in future research. This study suggested that MIR99AHG may serve as a novel target for GC patients undergoing ICIs therapy.

Seven mRNA genes were included in the final prognostic model, including *IL1RL, SPAG16, FAM110B, ANKRD6, ACSS3, CORO2B* and *TNFAIP8L3*. They were all downregulated in the MSIH group, positively correlated with MIR99AHG expression and were risk factors for the prognosis of GC. The Interleukin 1 Receptor Like 1 (*IL1RL1*) gene encodes for the Interleukin-33 (*IL-33*) receptor. A previous study showed that IL1RL1 was upregulated in GC tissues and IL-33/ IL1RL1 contributes to the progression of GC by regulating the MAPK pathway [15]. In addition, Mast cells can be activated by IL-33 and release macrophage-attracting factors to promote the accumulation of tumor-associated macrophages, then promote the progression of gastric cancer [15]. IL33/ IL1RL1 signaling can also promote the accumulation of tumor-infiltrating regulatory T-cells in the TME, suggesting that IL1RL1 may be a novel target for cancer immunotherapy [17]. The Sperm Associated Antigen 16 (*SPAG16*) is one of the cancer-testis (CT) antigens which are significantly associated with activation of the anticancer immune response. In a previous study, SPAG16 was upregulated in a variety of cancer tissues and may therefore serve as a potential target for immunotherapy [18]. The Family with Sequence Similarity 110 Member B (FAM110B) is located in the centrosome and its dysregulation is associated with abnormal cell cycle progression [19]. A recent study demonstrated that FAM110B was an immune-related hub gene in colorectal cancer and its expression was positively associated with the infiltration of multiple immune cells, such as CD4 T cells, macrophages, neutrophils, and dendritic cells [20]. Nonetheless, its role in GC remains unclear and warrants further research. Coronin 2B (CORO2B) is involved in a variety of cell adhesion and cytoskeletal related pathways [21]. It was identified as a TME-related gene and was associated with prognosis in esophageal squamous cell carcinoma [22]. In addition, the Ankyrin Repeat Domain 6 (ANKRD6), also known as Diversin, was highly expressed in several cancer tissues [23]. Rui Bai et al. reported that high expression of ANKRD6 was correlated with poor prognosis and high M2 Macrophage infiltration in colorectal cancer [24]. The tumor necrosis factor alpha-induced protein 8-like 3 (TNFAIP8L3) is a member of the death effector domain (DED)-containing protein coding gene family. It is involved in multiple biological processes such as immune homeostasis, inflammatory response, and tumorigenesis [25]. Additionally, TNFAIP8L3 can promote tumor invasion and metastasis of GC through the PI3K/Akt pathway and was related to poor prognosis [26]. Another study reported that miR-9-5p inhibits GC growth by downregulating TNFAIP8L3, suggesting its pivotal role in GC progression [27]. Moreover, the Acyl-CoA Synthetase Short Chain Family Member 3 (ACSS3) is recognized as a crucial regulator of cancer cell metabolism, especially in the context of metabolic stress [28]. According to previous research, ACSS3 exhibited high expression in GC and was correlated with poor survival. Depletion of ACSS3 inhibited the growth and invasion of GC cells, particularly under starvation [29]. In conclusion, all the seven target genes used for constructing the model play a critical role in cancer progression. Moreover, a review of existing literature confirmed the effect of these genes on the prognosis of cancer patients, consistent with our study. Importantly, the results demonstrated that several genes were involved in immune cell infiltration and immune response in the TME. These findings explain why the established prognosis predictors are also related to the efficacy of ICI in GC, indicating that the target genes in the hub MSI-related ceRNA network based on MIR99AHG may be future auxiliary targets for ICIs therapy in GC.

Immune cells in the TME regulates cancer development and progression, thereby influencing the prognosis of GC patients [30]. In this study, the relationship between the novel predictor and immune cell infiltration was analyzed. Results showed that among 22 types of immune cells analyzed, 8 immune cells were associated with risk score. Moreover, T cells CD4 memory resting, mast cells resting, B cells naïve and monocytes were enriched in the high-risk group. The other four cell types were enriched into the low-risk group, namely the T cells CD4 memory activated, mast cells activated, neutrophils and macrophages M1. Particularly, mast cells resting were identified as a risk factor whereas T cells CD4 memory activated were considered as a protective factor in our survival analysis. Evidence from prior studies has shown that the proportion of mast cells in GC tissues is higher than in normal tissues. Mast cells were identified as a risk factor in GC, because it promoted angiogenesis and metastasis of GC by releasing lymphangiogenic factors (*VEGF-C and VEGF-F*) and angiogenic factors (*VEGF-A, CXCL8, MMP-9*) [31]. In addition, studies have shown that programmed death ligands (*PD-L1 and PD-L2*) were expressed in mast cells infiltrating in the GC tissue. They can thus be used as an effective target for ICIs [31]. As for T cells CD4 memory activated, it was found that they mainly inhibit tumor growth. They release cytokines at an early stage to chemotactic the anti-tumor immune response, and enhance B cell and CD8+ T cell responses, and can directly kill damaged cells [32]. We further calculated immune, stromal, and the estimate score for evaluating the immune cell infiltration. The results indicated that the high-risk group had a higher TME score and lower tumor purity. Collectively, the variation in the content of these immune cells and immune functions between the two groups suggests a connection between the risk score and the TME. Chemokines are chemotactic cytokines which play a role in recruiting and locating immune cells in TME. In view of the plasticity of immune cells in the TME, we analyzed the differentially expressed immune cells and chemokines between the high- and low-risk groups, and the co-expression relationship among them. In total, we found 8 co-expression patterns between 6 chemokines and 5 immune cells (CX3CL1 ~ T cells CD4 memory activated, CXCL14 ~ B cells naïve, CXCL12 ~ mast cells resting, CXCL1 ~ neutrophils, CXCL2 ~ neutrophils, CXCL1 ~ mast cells activated, CXCL2 ~ mast cells activated and CCL19 ~ B cells naïve). Previously, Heqiang et al. found that *CagA- Helicobacter pylori* infection induced the release of CX3CL1 from the gastric epithelium which subsequently recruited CD4 T memory cells. This recruitment reduced the infection and damage caused by *H. pylori* [33]. The CXCL14, which is named as B cell and monocyte-activated chemokine (BMAC) was associated with the deterioration and metastasis of multiple malignant tumors [34]. As for CXCL12, Yipin et al. found that GC cells recruited mast cells by releasing CXCL12. Moreover, the recruited mast cells inhibited the immunotoxicity of T cells by increasing PD-L1 expression to promote immune evasion by GC cells [35]. CXCL1 and CXCL2 are important chemokines secreted by neutrophils. Studies have shown that CXCL1 and CXCL2 promoted the progression of GC by recruiting pro-tumoral neutrophils [36]. However, it has also been reported that CXCL1 and CXCL2 have bidirectional roles in cancer progression [37, 38]. In our study, the expression of CXCL1 and CXCL2 was significantly correlated with neutrophil infiltration. Moreover, both CXCL1 and CXCL2 were upregulated in the low-risk group, and the role of CXCL1 and CXCL2 in the pathogenesis of GC should be further investigated. CCL19 expression was significantly increased in the co-culture environment of *H. pylori*-infected gastric mucosa and B cell. *H. pylori* activates the NF-κB pathway in B cells to prevent apoptosis of B cells and promote the malignant conversion of B cells [39]. In addition, we analyzed the relationship among these 6 chemokines and MIR99AHG expression, and the results showed that MIR99AHG expression was positively correlated with CXCL14, CXCL12, and CCL19, and negatively correlated with CX3CL1, CXCL1 and CXCL2. Importantly, in our survival analysis, mast cells resting and MIR99AHG were identified as risk factors whereas T cells CD4 memory activated were considered as a protective factor. These results were consistent with each other and also with the previous literatures. Therefore, among the 8 co-expression patterns based on chemokines and immune cells, CXCL12 ~ mast cells resting and CX3CL1 ~ T cells CD4 memory activated should be further investigated.

## CONCLUSIONS

To summarize, our bioinformatic analyses revealed an MSI-related lncRNA-miRNA-mRNA network with the potential to predict the prognosis of GC. Performance of the prognosis prediction model was validated in external datasets, and was found to be associated with ICIs therapy response. The lncRNA MIR99AHG was negatively associated with PD-L1 and highly correlated with the expression of chemokine genes and immune cell infiltration in the GC TME. The established ceRNA network and prognostic model lays the foundation for future basic and clinical investigations, and MIR99AHG might be identified as a novel molecular target for accurate diagnosis, targeted therapy, and immunotherapy of GC patients.

## MATERIALS AND METHODS

### Research design and data preprocessing

The study was designed according to the flow diagram (Supplementary Figure 1).

Three GEO microarray datasets and the TCGA-STAD dataset were downloaded. Specifically, expression data from 689 samples and their corresponding clinical information were extracted from the GEO database (https://www.ncbi.nlm.nih.gov/geo/), including 200 samples from GSE15459, 56 from GSE34942 and 433 samples from GSE84437. The “Combat” function in the “sva” R package was used to remove the batch effect. Additionally, the mRNA, lncRNA and miRNA expression data from 348 patients and their clinical characteristics, were obtained from the TCGA GDC pipeline (https://portal.gdc.cancer.gov/). Moreover, immunophenoscore (IPS) and MSI data for patients in the TCGA database, were obtained from the Cancer Immunome Atlas (TCIA, https://tcia.at/home).

### Screening for differentially expressed mRNAs (DEmRNAs), miRNA (DEmiRNAs) and lncRNAs (DElncRNAs)

Referring to the revised Bethesda guidelines for colorectal cancer, samples were divided into two groups: samples displaying instability at two or more of the five recommended loci (*BAT-25, BAT-26, NR-21, NR-24, NR-27*) were interpreted as MSIH (19.2%), while tumors with only one or no locus altered were identified as MSIL/S (80.8%). The “gdcDEAnalysis” function in the “GDCRNATools” package was applied to screen for differentially expressed genes between the MSIH and MSIL/S groups [40]. The screening conditions were set at |log2 (fold change) |>1 and FDR<0.05 for mRNA and lncRNA, and |log2 (fold change) |>0.5 and FDR<0.05 for miRNA. A volcano plot was used to display the DEmRNAs, DEmiRNAs, and DElncRNAs.

### Construction of the lncRNA-miRNA-mRNA network

The “gdcCEAnalysis” function in the “GDCRNATools” package [16] was used to construct the ceRNA regulatory network based on the genes screened in step 2 (above). This function screens credible lncRNA-mRNA pairs based on the following criteria [41]: (1) a significant number of miRNAs must be shared between the lncRNA-mRNA pair (hypergeometric test, p<0.01); (2) a positive correlation must exist between this lncRNA-mRNA pair (Pearson correlation, Cor>0 and p<0.01); and (3) the common miRNAs should play similar roles in regulating the expression of the lncRNA and mRNA (regulation similarity>0) [42]. Notably, the database used to search for possible lncRNA -miRNA and mRNA-miRNA pairs can be set in the “gdcCEAnalysis” function. To explore as many pairs as possible, the parameter was set as “miRcode”, which constructed the largest network. Finally, the “clusterProfiler” package was used to conduct enrichment analysis targeting the mRNAs in the construed network, to reveal the potential functions and mechanisms of this ceRNA regulatory network.

### Screening for the hub ceRNA network with prognostic significance

The constructed ceRNA network from the previous step comprised of 2729 lncRNA-mRNA pairs and 895 nodes (808 mRNAs, 44miRNAs and 43 lncRNAs). Survival analysis was conducted on the 43 lncRNAs to identify the hub lncRNAs with prognostic significance. According to literature, lncRNAs exert their function based on their cellular position and sponging lncRNAs are mainly located in the cytoplasm [43]. Therefore, location prediction was conducted following survival analysis. In addition, nucleotide sequences for the lncRNAs of interest were downloaded from the NCBI nucleotide website (https://www.ncbi.nlm.nih.gov/nuccore/), then uploaded to the lncLocator database (http://www.csbio.sjtu.edu.cn/bioinf/lncLocator/) [44] to predict the possible positions of the lncRNAs. The result from this database revealed 5 possible locations and the corresponding possibility. Notably, MIR99AHG was targeted as the hub lncRNA, as it was located in the cytoplasm and was a significant risk factor for the overall survival of GC patients. Finally, we screened for the MIR99AHG-related mRNAs and miRNAs, then performed Unicox analysis on the related genes. Only genes with prognostic significance were retained and the final hub ceRNA regulatory network was constructed.

### Construction and validation of a prognosis prediction model

Based on the mRNAs in the final hub ceRNA regulatory network, the “glmnet” package was used to construct the prognosis prediction model. Thereafter, patients were stratified into the high- and low-risk groups based on the median value of the predicted risk score. In addition, survival differences between these 2 groups were analyzed using the Kaplan-Meier curve method. The efficiency of the model was tested through the ROC curve which was plotted using the “ROCR” package. Moreover, PCA analysis was conducted to test whether the predicted risk score could successfully stratify patients based on risk. MultiCox analysis was also conducted to show whether the predicted risk score was an independent prognostic factor in GC patients. To test the generalization ability of the established model, all the above analyses were conducted in the GEO datasets which have been detailed in the “Data Acquisition and Preparation” section. Furthermore, correlation analysis was conducted to check for consistencies between the risk score and existing clinical characteristics. Finally, differentially expressed genes between the 2 risk groups were screened using FDR≤0.05 and | logFCfilter | ≥1, and GO and KEGG enrichment analyses were conducted based on these differentially expressed genes.

### Correlation analysis between the predicted risk score and ICI response predictor

The IPS scores for 415 patients were downloaded from the TCIA database, which uses expression data and a machine learning-based algorithm to predict ICI (*PD1/PDL1/PDL2* blocker or *CTLA4* blocker) response in 20 types of solid tumors (range from 0-10, higher score means higher possibility to response to ICI) [10]. The downloaded TCGA-STAD IPS scores ranged from 4-10. Therefore, we divided patients into 3 different groups (4-6, 7-8 and 9-10) and the Wilcoxon rank test was used to show differences in the risk scores between the 3 categories. In addition, the study assessed the correlation between the hub genes in the final prognostic model and the *PD-L1/PD1/ CTLA4*.

### Analysis of immune cell infiltration between different risk groups

Basic data files for 22 types of immune cells and the R script for infiltration scoring were obtained from the CIBERSORT website (https://cibersort.stanford.edu/) [45]. The immune-cell infiltration score was calculated and compared between different risk groups. The immune, stromal, and the estimate score were calculated for each sample using the “estimate” package. And then we used “ggplot2” package to reveal differences in the immune score and stromal score between the high- and low-risk groups and the relationship between risk score, immune score, and the stromal score [46]. Notably, chemokines are a type of small cytokines with chemotactic functions. They guide cell migration and localization between tissues and play an important role in embryogenesis, tissue development and immune response [47]. Studies have shown that chemokines play an important role in immune cell infiltration in the tumor microenvironment (TME) [48]. Therefore, this study obtained the list of 40 human chemokines, and analyzed differences in their expression between different risk groups. Pearson correlation analysis was used to find the potential regulatory chemokine and immune cell pairs (denoted as “chemokine ~ immune cell”) based on the differentially expressed immune cells and chemokines between the 2 risk groups. Furthermore, the correlation between MIR99AHG and these chemokines was analyzed. Finally, patients were divided into two groups according to the median infiltration score of the immune cells of interest, and differences in survival between the two categories were analyzed using the KM curve.

### RNA isolation and qRT-PCR

Gastric cancer specimen and adjacent nontumor tissues were obtained from 15 individuals with GC who had undergone gastric cancer surgery between January 2021 and January 2022 in the Xijing Hospital of Digestive Diseases. The TRIzol reagent (Invitrogen, Waltham, MA, USA) was used to isolate total RNA from both GC and the adjacent nontumor tissues, according to the manufacturer’s protocol. Thereafter, 2 μg of the extracted total RNA was used for reverse transcription using the PrimeScript™ RT Master Mix kit (Takara). qRT-PCR analyses for the expression of the *MIR99AHG, IL1RL1, SPAG16, FAM110B, ANKRD6, ACSS3, CORO2B and TNFAIP8L3* genes were conducted on the CFX96 Real-Time System (Bio-Rad, Hercules, CA, USA), including with 45 cycles of 95° C for 5 s and 60° C for 30 s. The primers are listed in Supplementary Table 5. The 2^−ΔΔCt^ method was employed to analyze the relative changes in gene expression.

### Statistics

In this study, the Wilcoxon rank test was applied to compare indexes between the high and low risk groups. Pearson analysis was used to test for the correlation between variables of interest and the KM curve was employed for survival analysis. All the statistical analyses were conducted in R (Version 4.0.2 (2020-06-22)), and p<0.05 was considered to be statistically significant, unless otherwise stated. A Github page (https://github.com/itkwffc/MSI-STAD.git) were created and all scripts and supported data were uploaded.

## Supplementary Material

Supplementary Figures

Supplementary Table 1

Supplementary Table 2

Supplementary Table 3

Supplementary Table 4

Supplementary Table 5

## References

[1] Miller KD, Nogueira L, Devasia T, Mariotto AB, Yabroff KR, Jemal A, Kramer J, Siegel RL. Cancer treatment and survivorship statistics, 2022. CA Cancer J Clin. 2022; 72:409–36. 10.3322/caac.21731 35736631

[2] Choi S, Park S, Kim H, Kang SY, Ahn S, Kim KM. Gastric Cancer: Mechanisms, Biomarkers, and Therapeutic Approaches. Biomedicines. 2022; 10:543. 10.3390/biomedicines10030543 35327345 PMC8945014

[3] Puliga E, Corso S, Pietrantonio F, Giordano S. Microsatellite instability in Gastric Cancer: Between lights and shadows. Cancer Treat Rev. 2021; 95:102175. 10.1016/j.ctrv.2021.102175 33721595

[4] Giampieri R, Maccaroni E, Mandolesi A, Del Prete M, Andrikou K, Faloppi L, Bittoni A, Bianconi M, Scarpelli M, Bracci R, Scartozzi M, Cascinu S. Mismatch repair deficiency may affect clinical outcome through immune response activation in metastatic gastric cancer patients receiving first-line chemotherapy. Gastric Cancer. 2017; 20:156–63. 10.1007/s10120-016-0594-4 26796888

[5] Ratti M, Lampis A, Hahne JC, Passalacqua R, Valeri N. Microsatellite instability in gastric cancer: molecular bases, clinical perspectives, and new treatment approaches. Cell Mol Life Sci. 2018; 75:4151–62. 10.1007/s00018-018-2906-9 30173350 PMC6182336

[6] Salmena L, Poliseno L, Tay Y, Kats L, Pandolfi PP. A ceRNA hypothesis: the Rosetta Stone of a hidden RNA language? Cell. 2011; 146:353–8. 10.1016/j.cell.2011.07.014 21802130 PMC3235919

[7] Chan JJ, Tay Y. Noncoding RNA:RNA Regulatory Networks in Cancer. Int J Mol Sci. 2018; 19:1310. 10.3390/ijms19051310 29702599 PMC5983611

[8] Qi M, Yu B, Yu H, Li F. Integrated analysis of a ceRNA network reveals potential prognostic lncRNAs in gastric cancer. Cancer Med. 2020; 9:1798–817. 10.1002/cam4.2760 31923354 PMC7050084

[9] Zou D, Wang Y, Wang M, Zhao B, Hu F, Li Y, Zhang B. Bioinformatics analysis reveals the competing endogenous RNA (ceRNA) coexpression network in the tumor microenvironment and prognostic biomarkers in soft tissue sarcomas. Bioengineered. 2021; 12:496–506. 10.1080/21655979.2021.1879566 33587010 PMC8806339

[10] Charoentong P, Finotello F, Angelova M, Mayer C, Efremova M, Rieder D, Hackl H, Trajanoski Z. Pan-cancer Immunogenomic Analyses Reveal Genotype-Immunophenotype Relationships and Predictors of Response to Checkpoint Blockade. Cell Rep. 2017; 18:248–62. 10.1016/j.celrep.2016.12.019 28052254

[11] Yang B, Shen J, Xu L, Chen Y, Che X, Qu X, Liu Y, Teng Y, Li Z. Genome-Wide Identification of a Novel Eight-lncRNA Signature to Improve Prognostic Prediction in Head and Neck Squamous Cell Carcinoma. Front Oncol. 2019; 9:898. 10.3389/fonc.2019.00898 31620361 PMC6759597

[12] Ning P, Wu Z, Hu A, Li X, He J, Gong X, Xia Y, Shang Y, Bian H. Integrated genomic analyses of lung squamous cell carcinoma for identification of a possible competitive endogenous RNA network by means of TCGA datasets. PeerJ. 2018; 6:e4254. 10.7717/peerj.4254 29340250 PMC5768173

[13] Zhou L, Ma J. MIR99AHG/miR-204-5p/TXNIP/Nrf2/ARE Signaling Pathway Decreases Glioblastoma Temozolomide Sensitivity. Neurotox Res. 2022; 40:1152–62. 10.1007/s12640-022-00536-0 35904670

[14] Meng Q, Wang X, Xue T, Zhao Q, Wang W, Zhao K. Long noncoding RNA MIR99AHG promotes gastric cancer progression by inducing EMT and inhibiting apoptosis via miR577/FOXP1 axis. Cancer Cell Int. 2020; 20:414. 10.1186/s12935-020-01510-6 32874129 PMC7457246

[15] Huang N, Cui X, Li W, Zhang C, Liu L, Li J. IL-33/ST2 promotes the malignant progression of gastric cancer via the MAPK pathway. Mol Med Rep. 2021; 23:361. 10.3892/mmr.2021.12000 33760194 PMC7985998

[16] Ma Y, Wang N, Yang S. Skin cutaneous melanoma properties of immune-related lncRNAs identifying potential prognostic biomarkers. Aging (Albany NY). 2022; 14:3030–48. 10.18632/aging.203982 35361740 PMC9037265

[17] Son J, Cho JW, Park HJ, Moon J, Park S, Lee H, Lee J, Kim G, Park SM, Lira SA, Mckenzie AN, Kim HY, Choi CY, et al. Tumor-Infiltrating Regulatory T-cell Accumulation in the Tumor Microenvironment Is Mediated by IL33/ST2 Signaling. Cancer Immunol Res. 2020; 8:1393–406. 10.1158/2326-6066.CIR-19-0828 32878747

[18] Siliņa K, Zayakin P, Kalniņa Z, Ivanova L, Meistere I, Endzeliņš E, Abols A, Stengrēvics A, Leja M, Ducena K, Kozirovskis V, Linē A. Sperm-associated antigens as targets for cancer immunotherapy: expression pattern and humoral immune response in cancer patients. J Immunother. 2011; 34:28–44. 10.1097/CJI.0b013e3181fb64fa 21150711

[19] Hauge H, Patzke S, Aasheim HC. Characterization of the FAM110 gene family. Genomics. 2007; 90:14–27. 10.1016/j.ygeno.2007.03.002 17499476

[20] Wang X, Duanmu J, Fu X, Li T, Jiang Q. Analyzing and validating the prognostic value and mechanism of colon cancer immune microenvironment. J Transl Med. 2020; 18:324. 10.1186/s12967-020-02491-w 32859214 PMC7456375

[21] Rogg M, Yasuda-Yamahara M, Abed A, Dinse P, Helmstädter M, Conzelmann AC, Frimmel J, Sellung D, Biniossek ML, Kretz O, Grahammer F, Schilling O, Huber TB, Schell C. The WD40-domain containing protein CORO2B is specifically enriched in glomerular podocytes and regulates the ventral actin cytoskeleton. Sci Rep. 2017; 7:15910. 10.1038/s41598-017-15844-1 29162887 PMC5698439

[22] Zhang D, Qian C, Wei H, Qian X. Identification of the Prognostic Value of Tumor Microenvironment-Related Genes in Esophageal Squamous Cell Carcinoma. Front Mol Biosci. 2020; 7:599475. 10.3389/fmolb.2020.599475 33381521 PMC7767869

[23] Luan L, Zhao Y, Xu Z, Jiang G, Zhang X, Fan C, Liu D, Zhao H, Xu K, Wang M, Yu X, Wang E. Diversin increases the proliferation and invasion ability of non-small-cell lung cancer cells via JNK pathway. Cancer Lett. 2014; 344:232–8. 10.1016/j.canlet.2013.10.033 24246849

[24] Bai R, Wu D, Shi Z, Hu W, Li J, Chen Y, Ge W, Yuan Y, Zheng S. Pan-cancer analyses demonstrate that ANKRD6 is associated with a poor prognosis and correlates with M2 macrophage infiltration in colon cancer. Chin J Cancer Res. 2021; 33:93–102. 10.21147/j.issn.1000-9604.2021.01.10 33707932 PMC7941690

[25] Padmavathi G, Banik K, Monisha J, Bordoloi D, Shabnam B, Arfuso F, Sethi G, Fan L, Kunnumakkara AB. Novel tumor necrosis factor-α induced protein eight (TNFAIP8/TIPE) family: Functions and downstream targets involved in cancer progression. Cancer Lett. 2018; 432:260–71. 10.1016/j.canlet.2018.06.017 29920292

[26] Gao JF, Zhang H, Lv J, Fan YY, Feng D, Song L. Effects of the long and short isoforms of TIPE3 on the growth and metastasis of gastric cancer. Biomed Pharmacother. 2020; 124:109853. 10.1016/j.biopha.2020.109853 31978770

[27] Fan Y, Shi Y, Lin Z, Huang X, Li J, Huang W, Shen D, Zhuang G, Liu W. miR-9-5p Suppresses Malignant Biological Behaviors of Human Gastric Cancer Cells by Negative Regulation of TNFAIP8L3. Dig Dis Sci. 2019; 64:2823–9. 10.1007/s10620-019-05626-2 31140050

[28] Zhang J, Duan H, Feng Z, Han X, Gu C. Acetyl-CoA synthetase 3 promotes bladder cancer cell growth under metabolic stress. Oncogenesis. 2020; 9:46. 10.1038/s41389-020-0230-3 32398651 PMC7217873

[29] Chang WC, Cheng WC, Cheng BH, Chen L, Ju LJ, Ou YJ, Jeng LB, Yang MD, Hung YC, Ma WL. Mitochondrial Acetyl-CoA Synthetase 3 is Biosignature of Gastric Cancer Progression. Cancer Med. 2018; 7:1240–52. 10.1002/cam4.1295 29493120 PMC5911630

[30] Fridman WH, Pagès F, Sautès-Fridman C, Galon J. The immune contexture in human tumours: impact on clinical outcome. Nat Rev Cancer. 2012; 12:298–306. 10.1038/nrc3245 22419253

[31] Sammarco G, Varricchi G, Ferraro V, Ammendola M, De Fazio M, Altomare DF, Luposella M, Maltese L, Currò G, Marone G, Ranieri G, Memeo R. Mast Cells, Angiogenesis and Lymphangiogenesis in Human Gastric Cancer. Int J Mol Sci. 2019; 20:2106. 10.3390/ijms20092106 31035644 PMC6540185

[32] MacLeod MK, Clambey ET, Kappler JW, Marrack P. CD4 memory T cells: what are they and what can they do? Semin Immunol. 2009; 21:53–61. 10.1016/j.smim.2009.02.006 19269850 PMC2679806

[33] Sun H, He T, Wu Y, Yuan H, Ning J, Zhang Z, Deng X, Li B, Wu C. Cytotoxin-Associated Gene A-Negative *Helicobacter pylori* Promotes Gastric Mucosal CX3CR1^+^CD4^+^ Effector Memory T Cell Recruitment in Mice. Front Microbiol. 2022; 13:813774. 10.3389/fmicb.2022.813774 35154057 PMC8829513

[34] Westrich JA, Vermeer DW, Colbert PL, Spanos WC, Pyeon D. The multifarious roles of the chemokine CXCL14 in cancer progression and immune responses. Mol Carcinog. 2020; 59:794–806. 10.1002/mc.23188 32212206 PMC7282946

[35] Lv Y, Zhao Y, Wang X, Chen N, Mao F, Teng Y, Wang T, Peng L, Zhang J, Cheng P, Liu Y, Kong H, Chen W, et al. Increased intratumoral mast cells foster immune suppression and gastric cancer progression through TNF-α-PD-L1 pathway. J Immunother Cancer. 2019; 7:54. 10.1186/s40425-019-0530-3 30808413 PMC6390584

[36] Verbeke H, Geboes K, Van Damme J, Struyf S. The role of CXC chemokines in the transition of chronic inflammation to esophageal and gastric cancer. Biochim Biophys Acta. 2012; 1825:117–29. 10.1016/j.bbcan.2011.10.008 22079531

[37] Jia SN, Han YB, Yang R, Yang ZC. Chemokines in colon cancer progression. Semin Cancer Biol. 2022; 86:400–7. 10.1016/j.semcancer.2022.02.007 35183412

[38] Urbantat RM, Vajkoczy P, Brandenburg S. Advances in Chemokine Signaling Pathways as Therapeutic Targets in Glioblastoma. Cancers (Basel). 2021; 13:2983. 10.3390/cancers13122983 34203660 PMC8232256

[39] Ohmae T, Hirata Y, Maeda S, Shibata W, Yanai A, Ogura K, Yoshida H, Kawabe T, Omata M. Helicobacter pylori activates NF-kappaB via the alternative pathway in B lymphocytes. J Immunol. 2005; 175:7162–9. 10.4049/jimmunol.175.11.7162 16301619

[40] Boland CR, Thibodeau SN, Hamilton SR, Sidransky D, Eshleman JR, Burt RW, Meltzer SJ, Rodriguez-Bigas MA, Fodde R, Ranzani GN, Srivastava S. A National Cancer Institute Workshop on Microsatellite Instability for cancer detection and familial predisposition: development of international criteria for the determination of microsatellite instability in colorectal cancer. Cancer Res. 1998; 58:5248–57. 9823339

[41] Paci P, Colombo T, Farina L. Computational analysis identifies a sponge interaction network between long non-coding RNAs and messenger RNAs in human breast cancer. BMC Syst Biol. 2014; 8:83. 10.1186/1752-0509-8-83 25033876 PMC4113672

[42] Wang P, Zhang X, Sun N, Zhao Z, He J. Comprehensive Analysis of the Tumor Microenvironment in Cutaneous Melanoma associated with Immune Infiltration. J Cancer. 2020; 11:3858–70. 10.7150/jca.44413 32328190 PMC7171484

[43] Chen J, Song Y, Li M, Zhang Y, Lin T, Sun J, Wang D, Liu Y, Guo J, Yu W. Comprehensive analysis of ceRNA networks reveals prognostic lncRNAs related to immune infiltration in colorectal cancer. BMC Cancer. 2021; 21:255. 10.1186/s12885-021-07995-2 33750326 PMC7941714

[44] Cao Z, Pan X, Yang Y, Huang Y, Shen HB. The lncLocator: a subcellular localization predictor for long non-coding RNAs based on a stacked ensemble classifier. Bioinformatics. 2018; 34:2185–94. 10.1093/bioinformatics/bty085 29462250

[45] Newman AM, Liu CL, Green MR, Gentles AJ, Feng W, Xu Y, Hoang CD, Diehn M, Alizadeh AA. Robust enumeration of cell subsets from tissue expression profiles. Nat Methods. 2015; 12:453–7. 10.1038/nmeth.3337 25822800 PMC4739640

[46] Yoshihara K, Shahmoradgoli M, Martínez E, Vegesna R, Kim H, Torres-Garcia W, Treviño V, Shen H, Laird PW, Levine DA, Carter SL, Getz G, Stemke-Hale K, et al. Inferring tumour purity and stromal and immune cell admixture from expression data. Nat Commun. 2013; 4:2612. 10.1038/ncomms3612 24113773 PMC3826632

[47] Marcuzzi E, Angioni R, Molon B, Calì B. Chemokines and Chemokine Receptors: Orchestrating Tumor Metastasization. Int J Mol Sci. 2018; 20:96. 10.3390/ijms20010096 30591657 PMC6337330

[48] Kohli K, Pillarisetty VG, Kim TS. Key chemokines direct migration of immune cells in solid tumors. Cancer Gene Ther. 2022; 29:10–21. 10.1038/s41417-021-00303-x 33603130 PMC8761573

