# Supplementary material for: Identification of a novel MSI-related ceRNA network for predicting the prognosis and immunotherapy response of gastric cancer
Source: Aging (Albany NY). 2023 Jun 12;15(11):5164–89. doi: 10.18632/aging.204794 (PMC10292885; doi:10.18632/aging.204794)
Supplement: Supplementary Figures [file aging-15-204794-s001.pdf]

SUPPLEMENTARY FIGURES

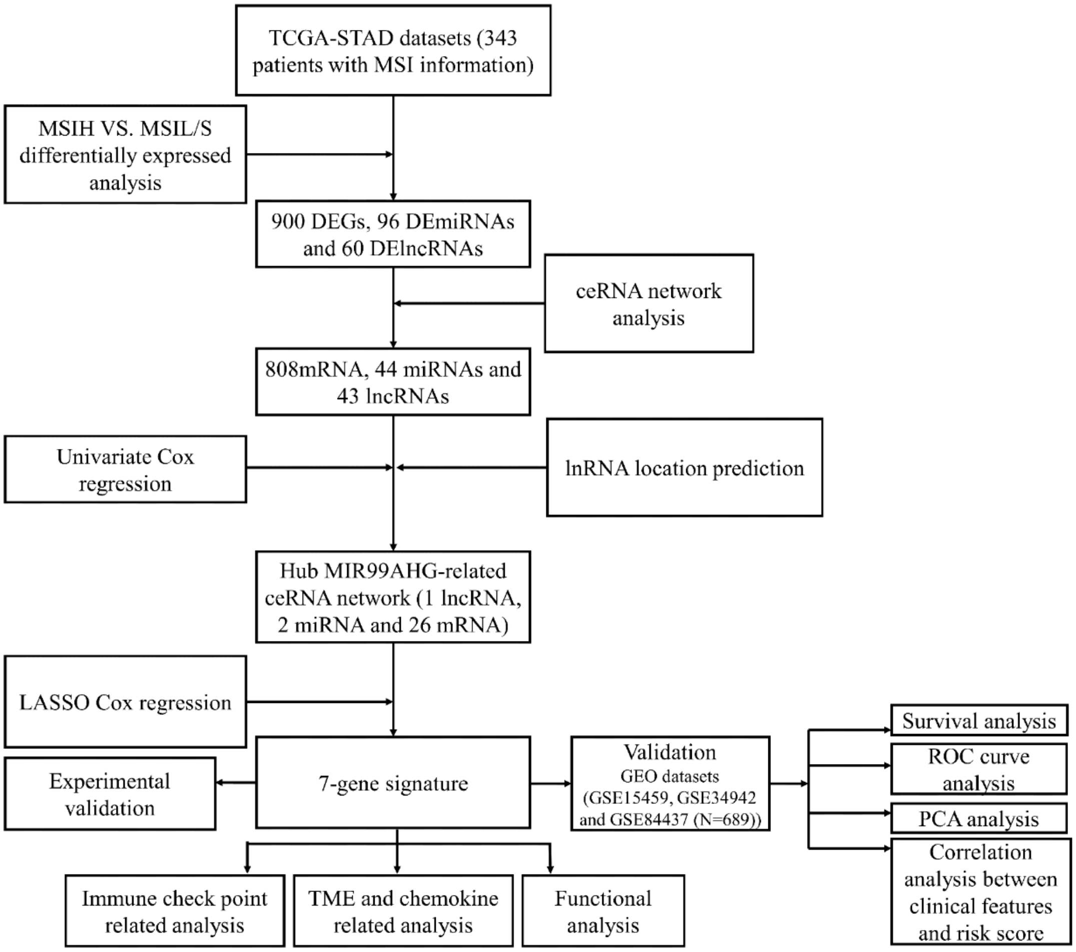

Supplementary Figure 1. The flow diagram of the study.

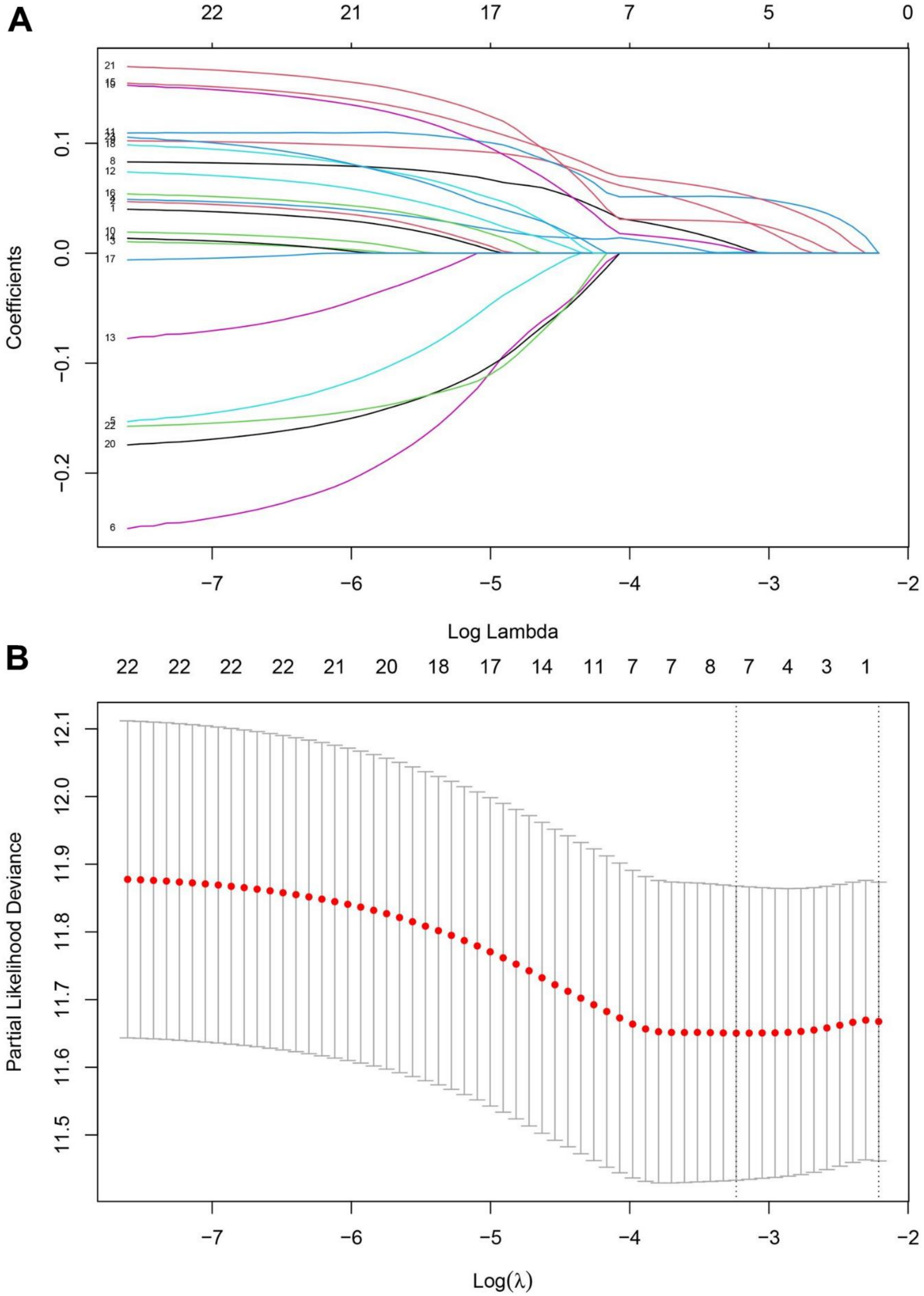

Supplementary Figure 2. Prognostic model constructed using LASSO algorithm. (**A**) LASSO coefficient profiles of the expression of 26 candidate RBPs. (**B**) Selection of the penalty parameter (λ) in the LASSO model via cross-validation.

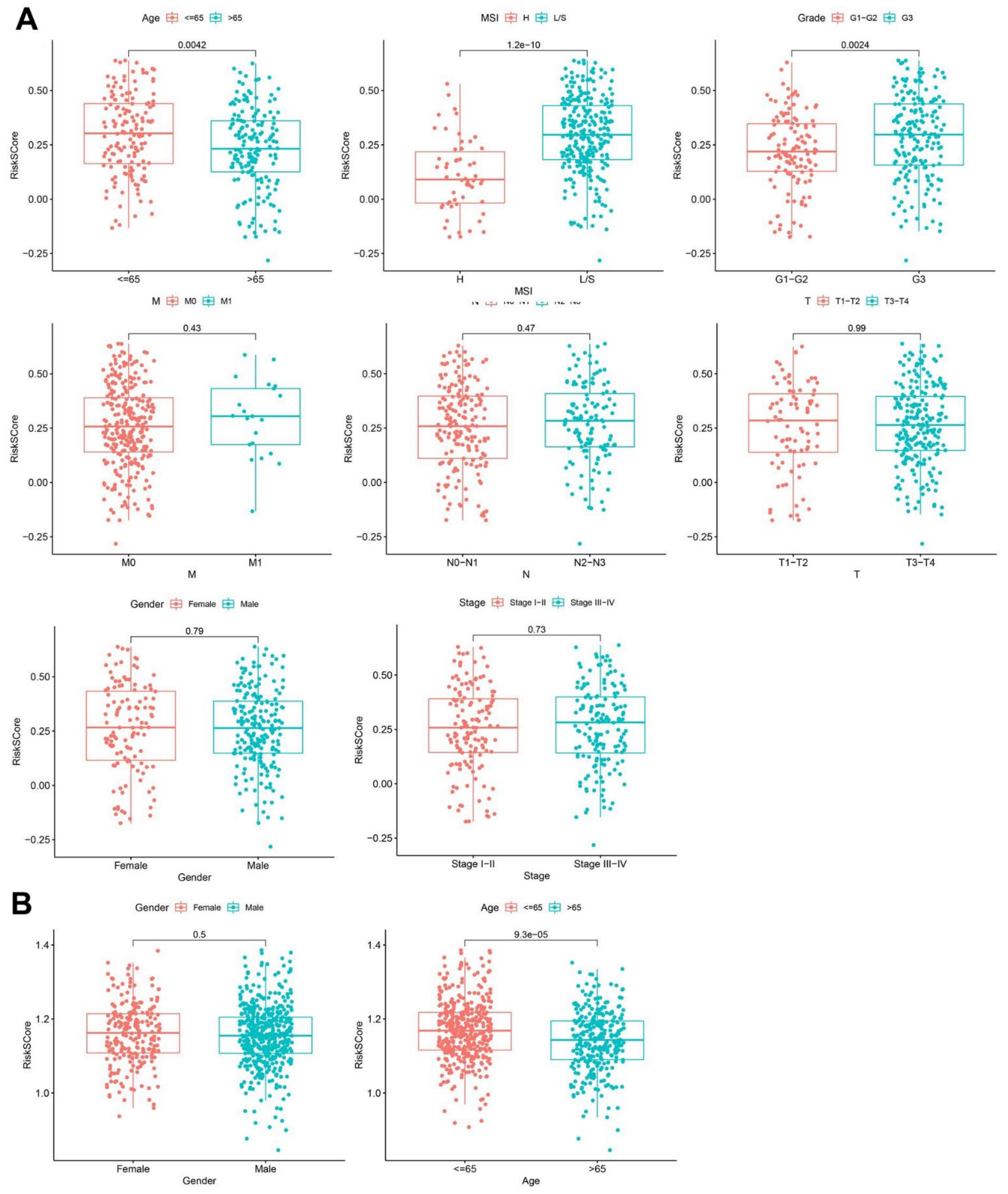

Supplementary Figure 3. Correlation between predicted risk score and clinical features. (**A**) TCGA database results. (**B**) GEO database results.

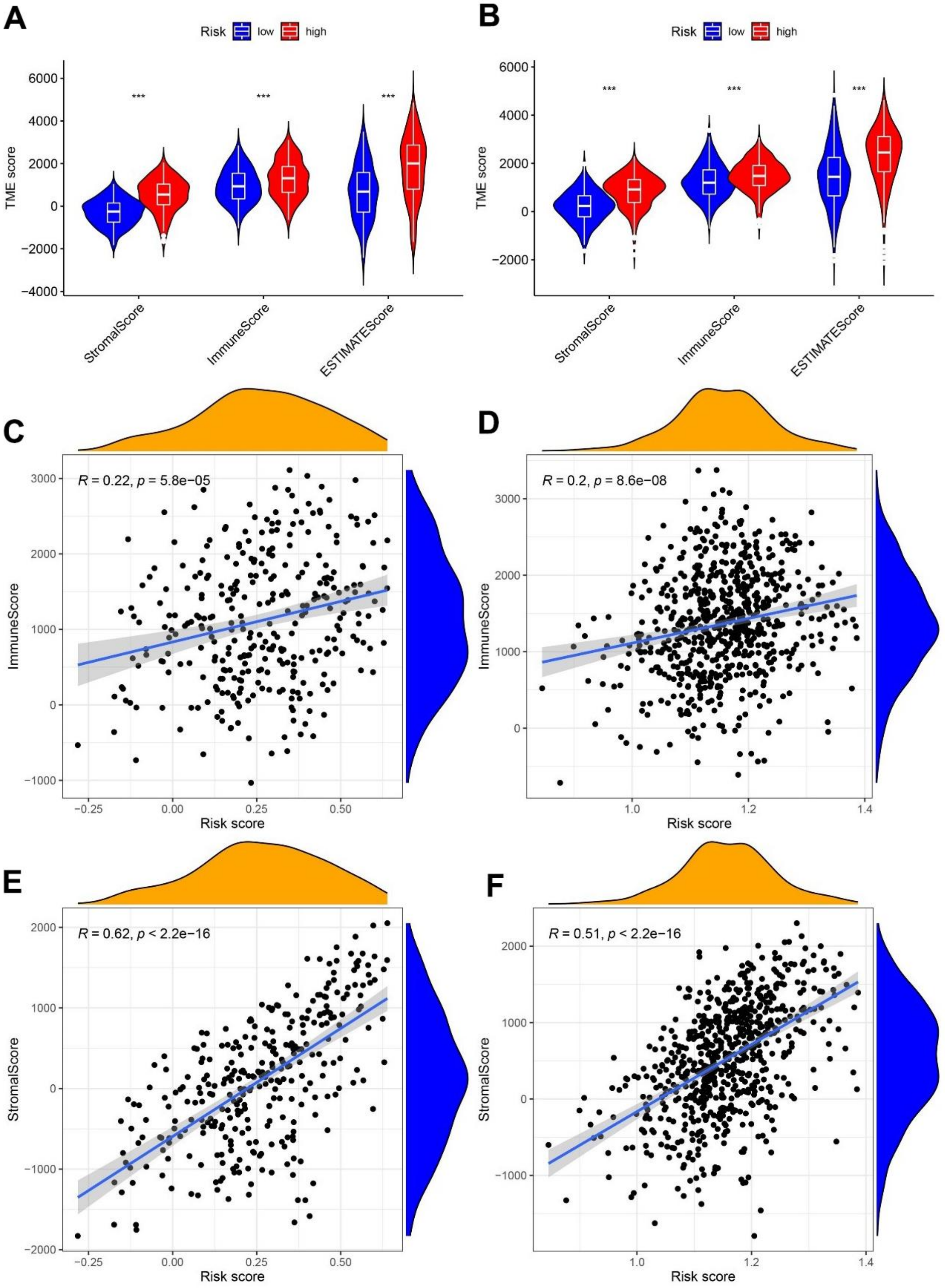

Supplementary Figure 4. Signature-related immune landscape based on the stromal, immune and ESTIMATE scores. (**A**, **B**) Differences in the TME score between the two risk groups in the TCGA and GEO datasets, respectively. (**C**, **D**) The relationship between the immune score and risk score in the TCGA and GEO datasets, respectively. (**E**, **F**) The relationship between the stromal score and risk score in the TCGA and GEO datasets, respectively.

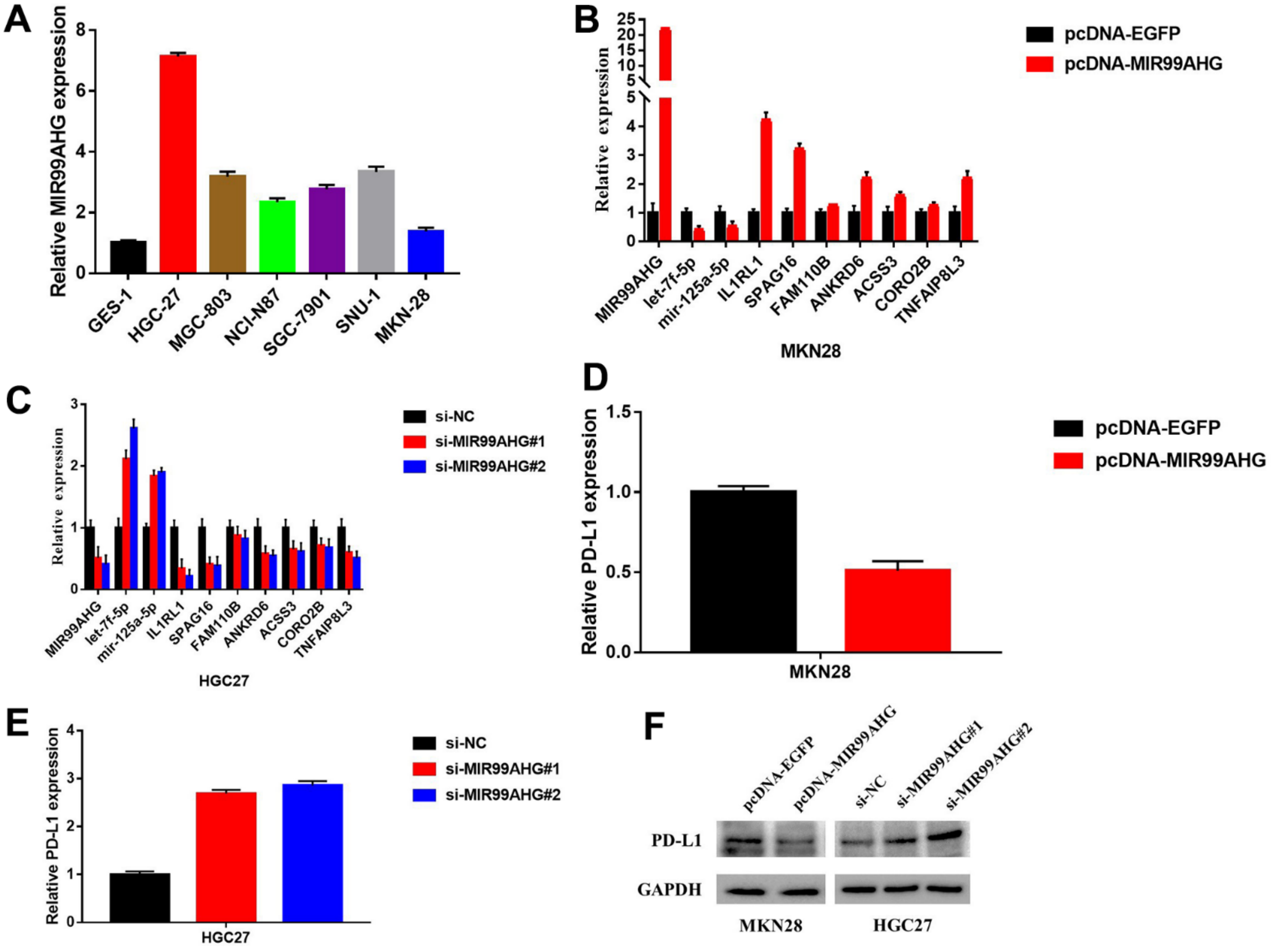

Supplementary Figure 5. Experimental validation. (**A**) Relative expression of MIR99AHG in six gastric cancer cell lines (hgc27, MGC803, NCI-N87, SGC7901, SNU-1 and MKN45) and human normal gastric epithelial cell line (GES1) analyzed by quantitative RT-PCR. (**B**, **C**) Effect of MIR99AHG overexpression or knockdown on the ceRNA network molecules expression. (**D**–**F**) Effect of MIR99AHG overexpression or knockdown on PD-L1 expression.
