# Supplementary material for: Identification of a novel MSI-related ceRNA network for predicting the prognosis and immunotherapy response of gastric cancer
Source: Aging (Albany NY). 2023 Jun 12;15(11):5164–89. doi: 10.18632/aging.204794 (PMC10292885; doi:10.18632/aging.204794)
Supplement: Supplementary Table 1 [file aging-15-204794-s002.pdf]

# Supplementary Tables

**Supplementary Table 1. Data information in TCGA and GEO database.**

| | TCGA overall (N=443) | | GEO overall (N=681) |
|---|---|---|---|
| **Event** | | **Fustat** | |
| Alive | 247 (55.8%) | 0 | 350 (51.4%) |
| Dead | 159 (35.9%) | 1 | 331 (48.6%) |
| Unknow | 37 (8.4%) | | |
| **Age** | | **Age** | |
| <=65 | 181 (40.9%) | <=65 | 384 (56.4%) |
| >65 | 222 (50.1%) | >65 | 297 (43.6%) |
| Unknow | 40 (9.0%) | **Gender** | |
| **Gender** | | Female | 224 (32.9%) |
| Female | 150 (33.9%) | Male | 457 (67.1%) |
| Male | 256 (57.8%) | | |
| Unknow | 37 (8.4%) | | |
| **Grade** | | | |
| G1 | 10 (2.3%) | | |
| G2 | 149 (33.6%) | | |
| G3 | 240 (54.2%) | | |
| Unknow | 44 (9.9%) | | |
| **Stage** | | | |
| Stage I | 56 (12.6%) | | |
| Stage II | 118 (26.6%) | | |
| Stage III | 167 (37.7%) | | |
| Stage IV | 39 (8.8%) | | |
| Unknow | 63 (14.2%) | | |
| **T** | | | |
| T1 | 23 (5.2%) | | |
| T2 | 85 (19.2%) | | |
| T3 | 185 (41.8%) | | |
| T4 | 103 (23.3%) | | |
| Unknow | 47 (10.6%) | | |
| **M** | | | |
| M0 | 361 (81.5%) | | |
| M1 | 27 (6.1%) | | |
| Unknow | 55 (12.4%) | | |
| **N** | | | |
| N0 | 122 (27.5%) | | |
| N1 | 109 (24.6%) | | |
| N2 | 80 (18.1%) | | |
| N3 | 78 (17.6%) | | |
| Unknow | 54 (12.2%) | | |
| **MSI** | | | |
| Stable | 295(66.6%) | | |
| Low | 63(14.2%) | | |
| High | 85 (19.2%) | | |
| **PD1_pos** | | | |
| 10 | 5 (1.1%) | | |
| 4 | 2 (0.5%) | | |
| 5 | 10 (2.3%) | | |
| 6 | 63 (14.2%) | | |
| 7 | 132 (29.8%) | | |
| 8 | 146 (33.0%) | | |
| 9 | 57 (12.9%) | | |
| Unknow | 28 (6.3%) | | |
| **CTLA4_pos** | | | |
| 10 | 23 (5.2%) | | |
| 5 | 2 (0.5%) | | |
| 6 | 26 (5.9%) | | |
| 7 | 83 (18.7%) | | |
| 8 | 157 (35.4%) | | |
| 9 | 124 (28.0%) | | |

| | |
|---|---|
| Unknow | 28 (6.3%) |
| **PD1_CTLA4_pos** | |
| 10 | 3 (0.7%) |
| 3 | 1 (0.2%) |
| 4 | 5 (1.1%) |
| 5 | 31 (7.0%) |
| 6 | 84 (19.0%) |
| 7 | 140 (31.6%) |
| 8 | 101 (22.8%) |
| 9 | 50 (11.3%) |
| Unknow | 28 (6.3%) |
