# Supplementary material for: Identification of a novel MSI-related ceRNA network for predicting the prognosis and immunotherapy response of gastric cancer
Source: Aging (Albany NY). 2023 Jun 12;15(11):5164–89. doi: 10.18632/aging.204794 (PMC10292885; doi:10.18632/aging.204794)
Supplement: Supplementary Table 2 [file aging-15-204794-s003.docx]

**Supplementary Table 2. MSI-related CeRNA.**

| lncRNAs | Genes | lncRNAscode | genecode | Counts | listTotal | popHits | popTotal | foldEnrichment | hyperPValue | deMIRCounts | cor | corPValue | regSim | sppc |
| --- | --- | --- | --- | --- | --- | --- | --- | --- | --- | --- | --- | --- | --- | --- |
| MIR99AHG | CBFA2T3 | ENSG00000215386 | ENSG00000129993 | 54 | 85 | 90 | 186 | 1.312941 | 0.000125 | 17 | 0.452181 | 2.13E-20 | 0.925572 | 0.054857 |
| MIR99AHG | CFAP221 | ENSG00000215386 | ENSG00000163075 | 62 | 85 | 108 | 186 | 1.256209 | 0.000129 | 18 | 0.301604 | 1.53E-09 | 0.885118 | 0.028277 |
| MIR99AHG | APBB1 | ENSG00000215386 | ENSG00000166313 | 50 | 85 | 82 | 186 | 1.33429 | 0.000172 | 17 | 0.622965 | 1.46E-41 | 0.92 | 0.066495 |
| MIR99AHG | SRPK3 | ENSG00000215386 | ENSG00000184343 | 49 | 85 | 80 | 186 | 1.340294 | 0.000183 | 11 | 0.221956 | 7.99E-06 | 0.774779 | 0.026764 |
| MIR99AHG | CACNG4 | ENSG00000215386 | ENSG00000075461 | 45 | 85 | 72 | 186 | 1.367647 | 0.00022 | 9 | 0.311612 | 4.25E-10 | 0.93215 | 0.029006 |
| MIR99AHG | RASD2 | ENSG00000215386 | ENSG00000100302 | 38 | 85 | 58 | 186 | 1.433671 | 0.000229 | 12 | 0.514373 | 9.52E-27 | 0.973657 | 0.056598 |
| MIR99AHG | ALPK3 | ENSG00000215386 | ENSG00000136383 | 69 | 85 | 126 | 186 | 1.198319 | 0.000245 | 20 | 0.413591 | 4.60E-17 | 0.83865 | 0.043889 |
| MIR99AHG | TMEM108 | ENSG00000215386 | ENSG00000144868 | 60 | 85 | 105 | 186 | 1.25042 | 0.000287 | 16 | 0.515586 | 6.94E-27 | 0.783285 | 0.037127 |
| MIR99AHG | C2orf40 | ENSG00000215386 | ENSG00000119147 | 31 | 85 | 45 | 186 | 1.507451 | 0.000304 | 4 | 0.761244 | 1.05E-71 | 0.967741 | 0.031927 |
| MIR99AHG | CPM | ENSG00000215386 | ENSG00000135678 | 68 | 85 | 124 | 186 | 1.2 | 0.000307 | 14 | 0.157515 | 0.001172 | 0.688882 | 0.00514 |
| MIR99AHG | GBGT1 | ENSG00000215386 | ENSG00000148288 | 49 | 85 | 81 | 186 | 1.323747 | 0.000312 | 11 | 0.321506 | 1.14E-10 | 0.856896 | 0.029235 |
| MIR99AHG | SEMA3E | ENSG00000215386 | ENSG00000170381 | 59 | 85 | 103 | 186 | 1.253455 | 0.000328 | 16 | 0.451918 | 2.25E-20 | 0.932203 | 0.034182 |
| MIR99AHG | TACR2 | ENSG00000215386 | ENSG00000075073 | 34 | 85 | 51 | 186 | 1.458824 | 0.000372 | 3 | 0.586855 | 5.21E-36 | 0.970588 | 0.038234 |
| MIR99AHG | FGF14 | ENSG00000215386 | ENSG00000102466 | 36 | 85 | 55 | 186 | 1.432299 | 0.000401 | 8 | 0.769635 | 3.39E-74 | 0.944202 | 0.023817 |
| MIR99AHG | IL1RL1 | ENSG00000215386 | ENSG00000115602 | 61 | 85 | 108 | 186 | 1.235948 | 0.000405 | 14 | 0.215164 | 1.46E-05 | 0.605863 | 0.008603 |
| MIR99AHG | NPHP1 | ENSG00000215386 | ENSG00000144061 | 77 | 85 | 148 | 186 | 1.138474 | 0.000452 | 21 | 0.282747 | 1.50E-08 | 0.869658 | 0.026322 |
| MIR99AHG | ZNF568 | ENSG00000215386 | ENSG00000198453 | 51 | 85 | 86 | 186 | 1.297674 | 0.000452 | 12 | 0.639519 | 2.34E-44 | 0.656803 | 0.021332 |
| MIR99AHG | FGF13 | ENSG00000215386 | ENSG00000129682 | 31 | 85 | 46 | 186 | 1.47468 | 0.000591 | 3 | 0.55767 | 5.25E-32 | 0.838707 | 0.028671 |
| MIR99AHG | CTTNBP2 | ENSG00000215386 | ENSG00000077063 | 67 | 85 | 123 | 186 | 1.191966 | 0.000607 | 18 | 0.46994 | 4.42E-22 | 0.940299 | 0.031362 |
| MIR99AHG | ADAMTS8 | ENSG00000215386 | ENSG00000134917 | 52 | 85 | 89 | 186 | 1.278519 | 0.000681 | 15 | 0.639813 | 2.08E-44 | 0.961538 | 0.041483 |
| MIR99AHG | MPP2 | ENSG00000215386 | ENSG00000108852 | 52 | 85 | 89 | 186 | 1.278519 | 0.000681 | 11 | 0.47161 | 3.04E-22 | 0.807692 | 0.041502 |
| MIR99AHG | BCAM | ENSG00000215386 | ENSG00000187244 | 34 | 85 | 52 | 186 | 1.430769 | 0.000686 | 12 | 0.31618 | 2.33E-10 | 0.882319 | 0.031259 |
| MIR99AHG | RTL5 | ENSG00000215386 | ENSG00000242732 | 34 | 85 | 52 | 186 | 1.430769 | 0.000686 | 7 | 0.67599 | 3.65E-51 | 0.941176 | 0.039376 |
| MIR99AHG | C15orf59 | ENSG00000215386 | ENSG00000205363 | 15 | 85 | 18 | 186 | 1.823529 | 0.000723 | 3 | 0.771529 | 8.98E-75 | 0.933333 | 0.037292 |
| MIR99AHG | OBSL1 | ENSG00000215386 | ENSG00000124006 | 47 | 85 | 79 | 186 | 1.301862 | 0.000954 | 13 | 0.437101 | 4.81E-19 | 0.759037 | 0.032838 |
| MIR99AHG | PTN | ENSG00000215386 | ENSG00000105894 | 16 | 85 | 20 | 186 | 1.750588 | 0.001088 | 3 | 0.705945 | 1.55E-57 | 0.9375 | 0.05034 |
| MIR99AHG | CAPN6 | ENSG00000215386 | ENSG00000077274 | 43 | 85 | 71 | 186 | 1.325269 | 0.001137 | 13 | 0.419917 | 1.40E-17 | 0.906943 | 0.023056 |
| MIR99AHG | SOBP | ENSG00000215386 | ENSG00000112320 | 56 | 85 | 99 | 186 | 1.23779 | 0.001182 | 13 | 0.560114 | 2.51E-32 | 0.946429 | 0.054954 |
| MIR99AHG | ANKRD6 | ENSG00000215386 | ENSG00000135299 | 70 | 85 | 132 | 186 | 1.160428 | 0.001292 | 16 | 0.549721 | 5.54E-31 | 0.871191 | 0.034464 |
| MIR99AHG | CLSTN2 | ENSG00000215386 | ENSG00000158258 | 55 | 85 | 97 | 186 | 1.240752 | 0.001306 | 8 | 0.493821 | 1.67E-24 | 0.865721 | 0.025178 |
| MIR99AHG | KIAA1644 | ENSG00000215386 | ENSG00000138944 | 59 | 85 | 106 | 186 | 1.21798 | 0.001316 | 16 | 0.551843 | 2.97E-31 | 0.932203 | 0.053181 |
| MIR99AHG | AKAP2 | ENSG00000215386 | ENSG00000241978 | 59 | 85 | 106 | 186 | 1.21798 | 0.001316 | 14 | 0.482543 | 2.46E-23 | 0.915254 | 0.040926 |
| MIR99AHG | LONRF2 | ENSG00000215386 | ENSG00000170500 | 78 | 85 | 153 | 186 | 1.115571 | 0.001377 | 19 | 0.510109 | 2.86E-26 | 0.897436 | 0.049956 |
| MIR99AHG | FIGN | ENSG00000215386 | ENSG00000182263 | 78 | 85 | 153 | 186 | 1.115571 | 0.001377 | 18 | 0.58347 | 1.59E-35 | 0.740559 | 0.026482 |
| MIR99AHG | SCN4B | ENSG00000215386 | ENSG00000177098 | 54 | 85 | 95 | 186 | 1.243839 | 0.001434 | 14 | 0.724297 | 7.46E-62 | 0.981481 | 0.046932 |
| MIR99AHG | ACSS3 | ENSG00000215386 | ENSG00000111058 | 58 | 85 | 104 | 186 | 1.220362 | 0.001479 | 13 | 0.616702 | 1.51E-40 | 0.896071 | 0.032016 |
| MIR99AHG | BMF | ENSG00000215386 | ENSG00000104081 | 47 | 85 | 80 | 186 | 1.285588 | 0.001527 | 11 | 0.13986 | 0.003487 | 0.863162 | 0.013129 |
| MIR99AHG | RGS5 | ENSG00000215386 | ENSG00000143248 | 53 | 85 | 93 | 186 | 1.247059 | 0.001566 | 17 | 0.544251 | 2.70E-30 | 0.817639 | 0.045813 |
| MIR99AHG | ZBTB16 | ENSG00000215386 | ENSG00000109906 | 57 | 85 | 102 | 186 | 1.222837 | 0.00165 | 12 | 0.670899 | 3.72E-50 | 0.964912 | 0.051319 |
| MIR99AHG | HLF | ENSG00000215386 | ENSG00000108924 | 66 | 85 | 123 | 186 | 1.174175 | 0.001776 | 16 | 0.513376 | 1.23E-26 | 0.787663 | 0.04198 |
| MIR99AHG | FLRT2 | ENSG00000215386 | ENSG00000185070 | 60 | 85 | 109 | 186 | 1.204533 | 0.001794 | 20 | 0.757165 | 1.57E-70 | 0.883333 | 0.035386 |
| MIR99AHG | C7 | ENSG00000215386 | ENSG00000112936 | 60 | 85 | 109 | 186 | 1.204533 | 0.001794 | 16 | 0.742786 | 1.43E-66 | 0.94365 | 0.035762 |
| MIR99AHG | CAPS | ENSG00000215386 | ENSG00000105519 | 42 | 85 | 70 | 186 | 1.312941 | 0.001903 | 9 | 0.160598 | 0.000958 | 0.833326 | 0.022488 |
| MIR99AHG | GGT7 | ENSG00000215386 | ENSG00000131067 | 31 | 85 | 48 | 186 | 1.413235 | 0.001957 | 13 | 0.132429 | 0.005334 | 0.794589 | 0.01908 |
| MIR99AHG | GFRA1 | ENSG00000215386 | ENSG00000151892 | 70 | 85 | 133 | 186 | 1.151703 | 0.002005 | 19 | 0.643302 | 5.08E-45 | 0.928493 | 0.04345 |
| MIR99AHG | DACT3 | ENSG00000215386 | ENSG00000197380 | 39 | 85 | 64 | 186 | 1.333456 | 0.002051 | 5 | 0.764338 | 1.30E-72 | 0.923077 | 0.017606 |
| MIR99AHG | COL4A5 | ENSG00000215386 | ENSG00000188153 | 65 | 85 | 121 | 186 | 1.175498 | 0.002087 | 18 | 0.448638 | 4.50E-20 | 0.889249 | 0.038132 |
| MIR99AHG | AKAP6 | ENSG00000215386 | ENSG00000151320 | 78 | 85 | 154 | 186 | 1.108327 | 0.002231 | 17 | 0.677631 | 1.71E-51 | 0.9345 | 0.04213 |
| MIR99AHG | LRRN1 | ENSG00000215386 | ENSG00000175928 | 48 | 85 | 83 | 186 | 1.265485 | 0.002255 | 13 | 0.333899 | 2.06E-11 | 0.791667 | 0.025614 |
| MIR99AHG | PLIN4 | ENSG00000215386 | ENSG00000167676 | 47 | 85 | 81 | 186 | 1.269717 | 0.002392 | 13 | 0.601044 | 4.16E-38 | 0.87234 | 0.043854 |
| MIR99AHG | ITIH5 | ENSG00000215386 | ENSG00000123243 | 73 | 85 | 141 | 186 | 1.132916 | 0.00246 | 20 | 0.668842 | 9.39E-50 | 0.917808 | 0.043946 |
| MIR99AHG | HOMER2 | ENSG00000215386 | ENSG00000103942 | 71 | 85 | 136 | 186 | 1.142388 | 0.002505 | 18 | 0.213078 | 1.75E-05 | 0.745029 | 0.026876 |
| MIR99AHG | CACHD1 | ENSG00000215386 | ENSG00000158966 | 52 | 85 | 92 | 186 | 1.236829 | 0.002614 | 15 | 0.13865 | 0.003742 | 0.563589 | 0.007836 |
| MIR99AHG | NOVA1 | ENSG00000215386 | ENSG00000139910 | 76 | 85 | 149 | 186 | 1.116147 | 0.002695 | 17 | 0.7165 | 5.61E-60 | 0.842027 | 0.029144 |
| MIR99AHG | GJC1 | ENSG00000215386 | ENSG00000182963 | 63 | 85 | 117 | 186 | 1.178281 | 0.002795 | 13 | 0.477407 | 8.10E-23 | 0.808696 | 0.044986 |
| MIR99AHG | SYNGR1 | ENSG00000215386 | ENSG00000100321 | 43 | 85 | 73 | 186 | 1.288961 | 0.002901 | 16 | 0.514096 | 1.02E-26 | 0.930233 | 0.059785 |
| MIR99AHG | CORO2B | ENSG00000215386 | ENSG00000103647 | 42 | 85 | 71 | 186 | 1.294449 | 0.003012 | 9 | 0.558536 | 4.05E-32 | 0.952381 | 0.044415 |
| MIR99AHG | MLH1 | ENSG00000215386 | ENSG00000076242 | 49 | 85 | 86 | 186 | 1.246785 | 0.003246 | 8 | 0.236663 | 2.03E-06 | 0.668434 | 0.01243 |
| MIR99AHG | CTSF | ENSG00000215386 | ENSG00000174080 | 39 | 85 | 65 | 186 | 1.312941 | 0.003286 | 9 | 0.519818 | 2.28E-27 | 0.923077 | 0.040849 |
| MIR99AHG | EFNB3 | ENSG00000215386 | ENSG00000108947 | 39 | 85 | 65 | 186 | 1.312941 | 0.003286 | 8 | 0.387211 | 5.09E-15 | 0.769118 | 0.020302 |
| MIR99AHG | ATP1B2 | ENSG00000215386 | ENSG00000129244 | 31 | 85 | 49 | 186 | 1.384394 | 0.00335 | 9 | 0.718805 | 1.59E-60 | 0.903226 | 0.042461 |
| MIR99AHG | TNFAIP8L3 | ENSG00000215386 | ENSG00000183578 | 37 | 85 | 61 | 186 | 1.32729 | 0.003407 | 9 | 0.693342 | 9.25E-55 | 0.999939 | 0.050111 |
| MIR99AHG | SPAG16 | ENSG00000215386 | ENSG00000144451 | 67 | 85 | 127 | 186 | 1.154423 | 0.003468 | 16 | 0.307739 | 7.02E-10 | 0.853812 | 0.019953 |
| MIR99AHG | MAPK10 | ENSG00000215386 | ENSG00000109339 | 67 | 85 | 127 | 186 | 1.154423 | 0.003468 | 16 | 0.800535 | 2.29E-84 | 0.940256 | 0.028186 |
| MIR99AHG | FAT4 | ENSG00000215386 | ENSG00000196159 | 78 | 85 | 155 | 186 | 1.101176 | 0.003566 | 20 | 0.623009 | 1.44E-41 | 0.910256 | 0.039729 |
| MIR99AHG | ROBO2 | ENSG00000215386 | ENSG00000185008 | 78 | 85 | 155 | 186 | 1.101176 | 0.003566 | 19 | 0.350285 | 1.89E-12 | 0.743584 | 0.004998 |
| MIR99AHG | PRELP | ENSG00000215386 | ENSG00000188783 | 52 | 85 | 93 | 186 | 1.223529 | 0.003943 | 13 | 0.823129 | 5.18E-93 | 1 | 0.031379 |
| MIR99AHG | PRICKLE2 | ENSG00000215386 | ENSG00000163637 | 66 | 85 | 125 | 186 | 1.155388 | 0.004059 | 18 | 0.689425 | 6.31E-54 | 0.939394 | 0.046322 |
| MIR99AHG | CLU | ENSG00000215386 | ENSG00000120885 | 56 | 85 | 102 | 186 | 1.201384 | 0.004162 | 14 | 0.425775 | 4.53E-18 | 0.855174 | 0.042254 |
| MIR99AHG | ZNF793 | ENSG00000215386 | ENSG00000188227 | 63 | 85 | 118 | 186 | 1.168295 | 0.004174 | 15 | 0.509365 | 3.46E-26 | 0.650679 | 0.028623 |
| MIR99AHG | PHYHD1 | ENSG00000215386 | ENSG00000175287 | 18 | 85 | 25 | 186 | 1.575529 | 0.004216 | 5 | 0.55548 | 1.01E-31 | 0.94425 | 0.03162 |
| MIR99AHG | FAM129A | ENSG00000215386 | ENSG00000135842 | 59 | 85 | 109 | 186 | 1.184458 | 0.004546 | 10 | 0.645401 | 2.16E-45 | 0.949153 | 0.045448 |
| MIR99AHG | VTN | ENSG00000215386 | ENSG00000109072 | 25 | 85 | 38 | 186 | 1.439628 | 0.004554 | 7 | 0.147337 | 0.002228 | 0.636278 | 0.010748 |
| MIR99AHG | AGT | ENSG00000215386 | ENSG00000135744 | 26 | 85 | 40 | 186 | 1.422353 | 0.004807 | 11 | 0.230986 | 3.48E-06 | 0.842578 | 0.017051 |
| MIR99AHG | CD1C | ENSG00000215386 | ENSG00000158481 | 39 | 85 | 66 | 186 | 1.293048 | 0.00513 | 10 | 0.455568 | 1.04E-20 | 0.974358 | 0.039101 |
| MIR99AHG | OGN | ENSG00000215386 | ENSG00000106809 | 38 | 85 | 64 | 186 | 1.299265 | 0.005258 | 10 | 0.82993 | 7.37E-96 | 0.947368 | 0.016543 |
| MIR99AHG | ERP27 | ENSG00000215386 | ENSG00000139055 | 29 | 85 | 46 | 186 | 1.37954 | 0.005345 | 5 | 0.15229 | 0.001638 | 0.550596 | -0.00859 |
| MIR99AHG | NMNAT2 | ENSG00000215386 | ENSG00000157064 | 53 | 85 | 96 | 186 | 1.208088 | 0.005404 | 14 | 0.248939 | 6.01E-07 | 0.810366 | 0.029979 |
| MIR99AHG | SUSD5 | ENSG00000215386 | ENSG00000173705 | 47 | 85 | 83 | 186 | 1.239121 | 0.005516 | 13 | 0.666931 | 2.20E-49 | 0.978723 | 0.035814 |
| MIR99AHG | CILP2 | ENSG00000215386 | ENSG00000160161 | 34 | 85 | 56 | 186 | 1.328571 | 0.005566 | 5 | 0.179937 | 0.000248 | 0.60487 | 0.007895 |
| MIR99AHG | CACNA1E | ENSG00000215386 | ENSG00000198216 | 72 | 85 | 141 | 186 | 1.117397 | 0.00704 | 18 | 0.192644 | 9.46E-05 | 0.691036 | 0.008237 |
| MIR99AHG | IL33 | ENSG00000215386 | ENSG00000137033 | 40 | 85 | 69 | 186 | 1.268542 | 0.007566 | 12 | 0.533805 | 5.15E-29 | 0.9 | 0.035671 |
| MIR99AHG | NRXN3 | ENSG00000215386 | ENSG00000021645 | 61 | 85 | 115 | 186 | 1.160716 | 0.007774 | 18 | 0.691834 | 1.94E-54 | 0.950678 | 0.041626 |
| MIR99AHG | FAM110B | ENSG00000215386 | ENSG00000169122 | 47 | 85 | 84 | 186 | 1.22437 | 0.008129 | 9 | 0.570799 | 9.30E-34 | 0.806331 | 0.037946 |
| MIR99AHG | P2RY1 | ENSG00000215386 | ENSG00000169860 | 26 | 85 | 41 | 186 | 1.387661 | 0.008144 | 5 | 0.37599 | 3.33E-14 | 0.730744 | 0.025965 |
| MIR99AHG | FBXL22 | ENSG00000215386 | ENSG00000197361 | 28 | 85 | 45 | 186 | 1.361569 | 0.008561 | 5 | 0.67494 | 5.91E-51 | 0.92857 | 0.025034 |
| MIR99AHG | PLN | ENSG00000215386 | ENSG00000198523 | 28 | 85 | 45 | 186 | 1.361569 | 0.008561 | 5 | 0.769467 | 3.81E-74 | 0.964263 | 0.035024 |

*** Only pairs associated with MIR99AHG were shown.**
