# Supplementary material for: Identification of a novel MSI-related ceRNA network for predicting the prognosis and immunotherapy response of gastric cancer
Source: Aging (Albany NY). 2023 Jun 12;15(11):5164–89. doi: 10.18632/aging.204794 (PMC10292885; doi:10.18632/aging.204794)
Supplement: Supplementary Table 3 [file aging-15-204794-s004.pdf]

Supplementary Table 3. Unicox results of hub CeRNA nodes.

Symbol	Type	HR	HR.95L	HR.95H	pvalue
MIR99AHG	lnc	1.110934	1.012531	1.218901	0.026207
hsa-let-7f-5p	mir	0.837702	0.718352	0.976882	0.023932
hsa-miR-125a-5p	mir	1.326392	1.078564	1.631165	0.007436
RASD2	pc	1.155149	1.012299	1.318157	0.032237
FGF14	pc	1.094469	1.001677	1.195856	0.04582
CORO2B	pc	1.169927	1.03789	1.318762	0.01021
EFNB3	pc	1.126146	1.011925	1.25326	0.029465
VTN	pc	1.085238	1.019612	1.155089	0.010163
MAPK10	pc	1.135742	1.015335	1.270427	0.026006
ACSS3	pc	1.190187	1.064229	1.331052	0.002283
C7	pc	1.07052	1.011043	1.133497	0.019463
IL1RL1	pc	1.107351	1.000791	1.225258	0.048236
ITIH5	pc	1.115472	1.002102	1.241667	0.045676
ATP1B2	pc	1.145148	1.022607	1.282374	0.01892
ANKRD6	pc	1.197949	1.043141	1.37573	0.010521
NOVA1	pc	1.102721	1.011909	1.201683	0.025751
RGS5	pc	1.193371	1.033213	1.378356	0.016202
SPAG16	pc	1.152548	1.019445	1.30303	0.023357
TMEM108	pc	1.17777	1.02045	1.359342	0.025306
GFRA1	pc	1.082308	1.009488	1.16038	0.026034
APBB1	pc	1.144415	1.004878	1.303327	0.04202
FAM110B	pc	1.173547	1.052473	1.308549	0.00397
SUSD5	pc	1.113545	1.009306	1.22855	0.031979
SCN4B	pc	1.118421	1.005903	1.243526	0.038569
TNFAIP8L3	pc	1.186751	1.053663	1.336649	0.004783
FLRT2	pc	1.177858	1.062291	1.305999	0.001891
FAT4	pc	1.138871	1.009026	1.285424	0.035251
PLN	pc	1.072955	1.001546	1.149454	0.045077
RTL5	pc	1.139186	1.008323	1.287034	0.036341
