# Supplementary material for: Identification of a novel MSI-related ceRNA network for predicting the prognosis and immunotherapy response of gastric cancer
Source: Aging (Albany NY). 2023 Jun 12;15(11):5164–89. doi: 10.18632/aging.204794 (PMC10292885; doi:10.18632/aging.204794)
Supplement: Supplementary Table 4 [file aging-15-204794-s005.docx]

Supplementary Table 4. KEGG enrichment results.

| Regarding genes between different risk groups | | | | | | | | Regarding genes among MSI-related ceRNA | | | | | | | |
| --- | --- | --- | --- | --- | --- | --- | --- | --- | --- | --- | --- | --- | --- | --- | --- |
| ID | **Description** | **GeneRatio** | **BgRatio** | **pvalue** | **p.adjust** | **qvalue** | **Count** | **ID** | **Description** | **GeneRatio** | **BgRatio** | **pvalue** | **p.adjust** | **qvalue** | **Count** |
| hsa04310 | Wnt signaling pathway | 16/366 | 167/8114 | 0.003489 | 0.032335 | 0.027457 | 16 | hsa04310 | Wnt signaling pathway | 18/366 | 167/8114 | 0.000509 | 0.012852 | 0.011436 | 18 |
| hsa04270 | Vascular smooth muscle contraction | 24/366 | 134/8114 | 5.06E-09 | 4.54E-07 | 3.85E-07 | 24 | hsa04270 | Vascular smooth muscle contraction | 15/366 | 134/8114 | 0.000995 | 0.021268 | 0.018925 | 15 |
| hsa00350 | Tyrosine metabolism | 5/366 | 36/8114 | 0.021661 | 0.132425 | 0.112449 | 5 | hsa00350 | Tyrosine metabolism | 8/366 | 36/8114 | 0.000158 | 0.005492 | 0.004887 | 8 |
| hsa04530 | Tight junction | 15/366 | 169/8114 | 0.009231 | 0.067112 | 0.056988 | 15 | hsa04530 | Tight junction | 13/366 | 169/8114 | 0.04169 | 0.289744 | 0.257818 | 13 |
| hsa04974 | Protein digestion and absorption | 12/366 | 103/8114 | 0.002222 | 0.025982 | 0.022063 | 12 | hsa04974 | Protein digestion and absorption | 20/366 | 103/8114 | 2.40E-08 | 6.67E-06 | 5.94E-06 | 20 |
| hsa04151 | PI3K-Akt signaling pathway | 31/366 | 354/8114 | 0.00028 | 0.004713 | 0.004002 | 31 | hsa04151 | PI3K-Akt signaling pathway | 25/366 | 354/8114 | 0.01706 | 0.148212 | 0.131882 | 25 |
| hsa04921 | Oxytocin signaling pathway | 16/366 | 154/8114 | 0.001522 | 0.018613 | 0.015806 | 16 | hsa04921 | Oxytocin signaling pathway | 13/366 | 154/8114 | 0.02161 | 0.176694 | 0.157225 | 13 |
| hsa05032 | Morphine addiction | 10/366 | 91/8114 | 0.007637 | 0.057067 | 0.048459 | 10 | hsa05032 | Morphine addiction | 10/366 | 91/8114 | 0.007637 | 0.084926 | 0.075569 | 10 |
| hsa04010 | MAPK signaling pathway | 24/366 | 294/8114 | 0.003453 | 0.032335 | 0.027457 | 24 | hsa04010 | MAPK signaling pathway | 23/366 | 294/8114 | 0.006947 | 0.081219 | 0.07227 | 23 |
| hsa04911 | Insulin secretion | 8/366 | 86/8114 | 0.039541 | 0.212098 | 0.180103 | 8 | hsa04911 | Insulin secretion | 11/366 | 86/8114 | 0.00158 | 0.02831 | 0.025191 | 11 |
| hsa05410 | Hypertrophic cardiomyopathy | 19/366 | 90/8114 | 1.28E-08 | 8.64E-07 | 7.33E-07 | 19 | hsa05410 | Hypertrophic cardiomyopathy | 16/366 | 90/8114 | 2.14E-06 | 0.000119 | 0.000106 | 16 |
| hsa04340 | Hedgehog signaling pathway | 8/366 | 56/8114 | 0.003379 | 0.032335 | 0.027457 | 8 | hsa04340 | Hedgehog signaling pathway | 6/366 | 56/8114 | 0.039332 | 0.287747 | 0.256042 | 6 |
| hsa04510 | Focal adhesion | 26/366 | 201/8114 | 1.05E-06 | 4.05E-05 | 3.44E-05 | 26 | hsa04510 | Focal adhesion | 22/366 | 201/8114 | 9.91E-05 | 0.003934 | 0.003501 | 22 |
| hsa04512 | ECM-receptor interaction | 20/366 | 88/8114 | 1.33E-09 | 1.79E-07 | 1.52E-07 | 20 | hsa04512 | ECM-receptor interaction | 16/366 | 88/8114 | 1.56E-06 | 0.000109 | 9.67E-05 | 16 |
| hsa05414 | Dilated cardiomyopathy | 21/366 | 96/8114 | 1.09E-09 | 1.79E-07 | 1.52E-07 | 21 | hsa05414 | Dilated cardiomyopathy | 17/366 | 96/8114 | 1.08E-06 | 0.0001 | 8.94E-05 | 17 |
| hsa04060 | Cytokine-cytokine receptor interaction | 24/366 | 295/8114 | 0.003606 | 0.032335 | 0.027457 | 24 | hsa04060 | Cytokine-cytokine receptor interaction | 20/366 | 295/8114 | 0.044442 | 0.294161 | 0.261749 | 20 |
| hsa04927 | Cortisol synthesis and secretion | 7/366 | 65/8114 | 0.026381 | 0.15427 | 0.130998 | 7 | hsa04927 | Cortisol synthesis and secretion | 8/366 | 65/8114 | 0.008454 | 0.090396 | 0.080436 | 8 |
| hsa04610 | Complement and coagulation cascades | 15/366 | 85/8114 | 4.90E-06 | 0.000165 | 0.00014 | 15 | hsa04610 | Complement and coagulation cascades | 12/366 | 85/8114 | 0.000396 | 0.011008 | 0.009795 | 12 |
| hsa04713 | Circadian entrainment | 13/366 | 97/8114 | 0.000386 | 0.006103 | 0.005183 | 13 | hsa04713 | Circadian entrainment | 10/366 | 97/8114 | 0.011787 | 0.121366 | 0.107993 | 10 |
| hsa04022 | cGMP-PKG signaling pathway | 24/366 | 167/8114 | 3.98E-07 | 2.14E-05 | 1.82E-05 | 24 | hsa04022 | cGMP-PKG signaling pathway | 16/366 | 167/8114 | 0.003489 | 0.051054 | 0.045428 | 16 |
| hsa04514 | Cell adhesion molecules | 22/366 | 149/8114 | 7.66E-07 | 3.43E-05 | 2.92E-05 | 22 | hsa04514 | Cell adhesion molecules | 23/366 | 149/8114 | 1.88E-07 | 2.61E-05 | 2.32E-05 | 23 |
| hsa04260 | Cardiac muscle contraction | 9/366 | 87/8114 | 0.016133 | 0.108494 | 0.092128 | 9 | hsa04260 | Cardiac muscle contraction | 9/366 | 87/8114 | 0.016133 | 0.147708 | 0.131433 | 9 |
| hsa04020 | Calcium signaling pathway | 21/366 | 240/8114 | 0.0027 | 0.030259 | 0.025694 | 21 | hsa04020 | Calcium signaling pathway | 21/366 | 240/8114 | 0.0027 | 0.044147 | 0.039283 | 21 |
| hsa05217 | Basal cell carcinoma | 7/366 | 63/8114 | 0.022619 | 0.135211 | 0.114814 | 7 | hsa05217 | Basal cell carcinoma | 8/366 | 63/8114 | 0.007012 | 0.081219 | 0.07227 | 8 |
| hsa04360 | Axon guidance | 19/366 | 182/8114 | 0.000535 | 0.007749 | 0.00658 | 19 | hsa04360 | Axon guidance | 20/366 | 182/8114 | 0.000194 | 0.005983 | 0.005323 | 20 |
| hsa05412 | Arrhythmogenic right ventricular cardiomyopathy | 14/366 | 77/8114 | 7.08E-06 | 0.000211 | 0.00018 | 14 | hsa05412 | Arrhythmogenic right ventricular cardiomyopathy | 14/366 | 77/8114 | 7.08E-06 | 0.000328 | 0.000292 | 14 |
| hsa04925 | Aldosterone synthesis and secretion | 12/366 | 98/8114 | 0.001444 | 0.018499 | 0.015709 | 12 | hsa04925 | Aldosterone synthesis and secretion | 10/366 | 98/8114 | 0.012619 | 0.125284 | 0.11148 | 10 |
| hsa04261 | Adrenergic signaling in cardiomyocytes | 13/366 | 150/8114 | 0.017761 | 0.116527 | 0.098949 | 13 | hsa04261 | Adrenergic signaling in cardiomyocytes | 15/366 | 150/8114 | 0.003073 | 0.047463 | 0.042233 | 15 |
| hsa05205 | Proteoglycans in cancer | 24/366 | 205/8114 | 1.57E-05 | 0.000423 | 0.000359 | 24 | hsa02010 | ABC transporters | 8/366 | 45/8114 | 0.000789 | 0.018287 | 0.016272 | 8 |
| hsa05146 | Amoebiasis | 15/366 | 102/8114 | 4.72E-05 | 0.001154 | 0.00098 | 15 | hsa00982 | Drug metabolism - cytochrome P450 | 10/366 | 72/8114 | 0.001357 | 0.026949 | 0.02398 | 10 |
| hsa05144 | Malaria | 10/366 | 50/8114 | 6.29E-05 | 0.001409 | 0.001197 | 10 | hsa04934 | Cushing syndrome | 16/366 | 155/8114 | 0.001629 | 0.02831 | 0.025191 | 16 |
| hsa04350 | TGF-beta signaling pathway | 14/366 | 94/8114 | 7.30E-05 | 0.001511 | 0.001283 | 14 | hsa00360 | Phenylalanine metabolism | 4/366 | 16/8114 | 0.004812 | 0.066884 | 0.059514 | 4 |
| hsa04640 | Hematopoietic cell lineage | 14/366 | 99/8114 | 0.00013 | 0.002493 | 0.002117 | 14 | hsa04926 | Relaxin signaling pathway | 13/366 | 129/8114 | 0.005319 | 0.070411 | 0.062653 | 13 |
| hsa04924 | Renin secretion | 11/366 | 69/8114 | 0.000234 | 0.004192 | 0.003559 | 11 | hsa04976 | Bile secretion | 10/366 | 89/8114 | 0.006541 | 0.081219 | 0.07227 | 10 |
| hsa04540 | Gap junction | 12/366 | 88/8114 | 0.000547 | 0.007749 | 0.00658 | 12 | hsa04724 | Glutamatergic synapse | 11/366 | 114/8114 | 0.013571 | 0.130096 | 0.115761 | 11 |
| hsa04810 | Regulation of actin cytoskeleton | 21/366 | 218/8114 | 0.000811 | 0.010901 | 0.009257 | 21 | hsa04930 | Type II diabetes mellitus | 6/366 | 46/8114 | 0.016471 | 0.147708 | 0.131433 | 6 |
| hsa04015 | Rap1 signaling pathway | 19/366 | 210/8114 | 0.002924 | 0.030758 | 0.026119 | 19 | hsa04614 | Renin-angiotensin system | 4/366 | 23/8114 | 0.018259 | 0.153817 | 0.136868 | 4 |
| hsa04970 | Salivary secretion | 11/366 | 93/8114 | 0.002973 | 0.030758 | 0.026119 | 11 | hsa04728 | Dopaminergic synapse | 11/366 | 132/8114 | 0.035814 | 0.28252 | 0.251391 | 11 |
| hsa04611 | Platelet activation | 13/366 | 124/8114 | 0.003794 | 0.032919 | 0.027953 | 13 | hsa05226 | Gastric cancer | 12/366 | 149/8114 | 0.036585 | 0.28252 | 0.251391 | 12 |
| hsa04730 | Long-term depression | 8/366 | 60/8114 | 0.005206 | 0.043767 | 0.037165 | 8 | hsa04916 | Melanogenesis | 9/366 | 101/8114 | 0.03811 | 0.28634 | 0.25479 | 9 |
| hsa05202 | Transcriptional misregulation in cancer | 17/366 | 193/8114 | 0.006226 | 0.050029 | 0.042482 | 17 | hsa04024 | cAMP signaling pathway | 16/366 | 221/8114 | 0.041491 | 0.289744 | 0.257818 | 16 |
| hsa04918 | Thyroid hormone synthesis | 9/366 | 75/8114 | 0.006323 | 0.050029 | 0.042482 | 9 | hsa01523 | Antifolate resistance | 4/366 | 30/8114 | 0.044206 | 0.294161 | 0.261749 | 4 |
| hsa04971 | Gastric acid secretion | 9/366 | 76/8114 | 0.006896 | 0.053002 | 0.045007 | 9 | hsa04727 | GABAergic synapse | 8/366 | 89/8114 | 0.046931 | 0.303412 | 0.26998 | 8 |
| hsa04062 | Chemokine signaling pathway | 16/366 | 192/8114 | 0.012963 | 0.091764 | 0.077921 | 16 |  |  |  |  |  |  |  |  |
| hsa04061 | Viral protein interaction with cytokine and cytokine receptor | 10/366 | 100/8114 | 0.014413 | 0.09941 | 0.084414 | 10 |  |  |  |  |  |  |  |  |
| hsa00590 | Arachidonic acid metabolism | 7/366 | 61/8114 | 0.019245 | 0.123262 | 0.104668 | 7 |  |  |  |  |  |  |  |  |
| hsa04977 | Vitamin digestion and absorption | 4/366 | 24/8114 | 0.021151 | 0.132319 | 0.112358 | 4 |  |  |  |  |  |  |  |  |
| hsa04014 | Ras signaling pathway | 17/366 | 232/8114 | 0.032948 | 0.188572 | 0.160126 | 17 |  |  |  |  |  |  |  |  |
| hsa04933 | AGE-RAGE signaling pathway in diabetic complications | 9/366 | 100/8114 | 0.036084 | 0.202223 | 0.171717 | 9 |  |  |  |  |  |  |  |  |
| hsa04392 | Hippo signaling pathway - multiple species | 4/366 | 29/8114 | 0.039672 | 0.212098 | 0.180103 | 4 |  |  |  |  |  |  |  |  |
| hsa04972 | Pancreatic secretion | 9/366 | 102/8114 | 0.040212 | 0.212098 | 0.180103 | 9 |  |  |  |  |  |  |  |  |
| hsa05165 | Human papillomavirus infection | 22/366 | 331/8114 | 0.043566 | 0.22537 | 0.191372 | 22 |  |  |  |  |  |  |  |  |

*Top 28 pathways were identical in enrichment results regarding genes between different risk groups and genes among MSI-related ceRNA.
