# Supplementary material for: Identification of a novel MSI-related ceRNA network for predicting the prognosis and immunotherapy response of gastric cancer
Source: Aging (Albany NY). 2023 Jun 12;15(11):5164–89. doi: 10.18632/aging.204794 (PMC10292885; doi:10.18632/aging.204794)
Supplement: Supplementary Table 5 [file aging-15-204794-s006.pdf]

Supplementary Table 5. Primers in quantitative real time-polymerase chain reaction.

Gene	Forward primer	Reverse primer
MIR99AHG	TAGCAAGGCCCAACCAGTTC	TCCCTTTGCAGCTCAGTAGT
IL1RL1	ATGGGGTTTTGGATCTTAGCAAT	CACGGTGTAACTAGGTTTTCCTT
SPAG16	ATGGGTGTTGGGACGAAGG	TGAAAGCAATCAAGAGTTCTGGT
FAM110B	TAGCTCCGAGGGCTCTAGC	CACCTTGCGGATGTCCGAA
ANKRD6	GTCGCTGCACTTTCAGAGC	CCATGCTTGGTAACCGCTAC
ACSS3	TGGACCAAAACGCTGGAGAAC	ACGATCAACGGCATTGTAACA
CORO2B	CGTCCGCAATACCGTAGCTC	TAGTTGGGTTCAATCCTGCCT
TNFAIP8L	AAGCACACTGGTTTCCACACT	TGGGTCCCTGCATATCCGTT
